# Accessing the Cloke-Wilson Rearrangement via Conjugate Addition of Phosphoranes to Michael Acceptors: A Route to Cyclopropanes and 5-Membered Ring Heterocycles Investigated by Density Functional and *Ab Initio* Theory

**DOI:** 10.1021/acs.joc.4c00757

**Published:** 2024-08-13

**Authors:** Götz Bucher

**Affiliations:** School of Chemistry, University of Glasgow, Joseph Black Building, University Avenue, Glasgow G12 8QQ, U.K.

## Abstract

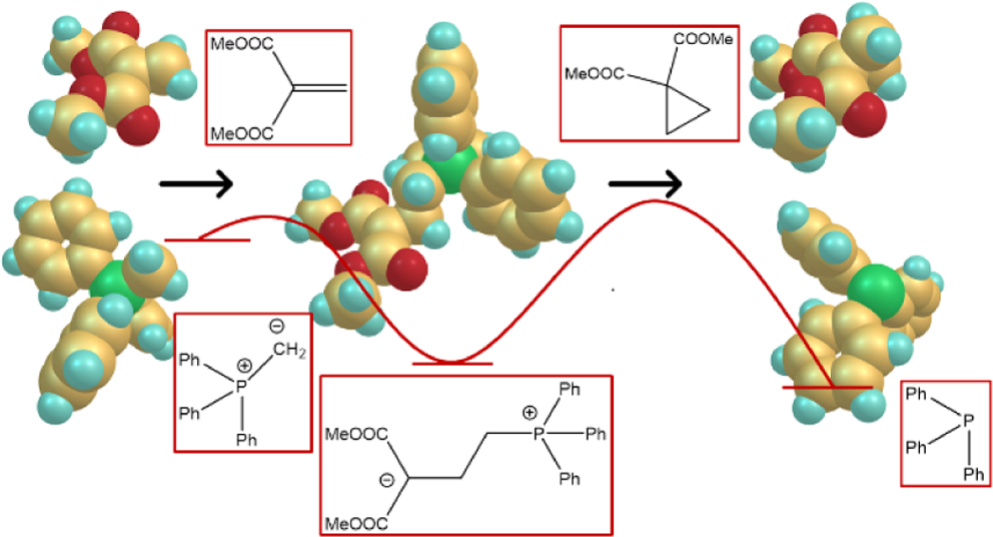

Conjugate addition of unstabilized Wittig-type phosphonium ylides to 1,1-diacceptor- and 1-acceptor-substituted alkenes is investigated by density functional theory and high-level *ab initio* (DLPNO–CCSD(T)) calculations. The results indicate that the initial conjugate addition step should be facile with barriers predicted to be between 0 and 21 kcal mol^–1^. Potential intramolecular follow-up reactions include the formation of acceptor-substituted cyclopropanes as well as the formation of dihydrofuran derivatives via intramolecular S_N_2-type transition state structures. The barriers calculated for these potentially valuable cyclization reactions are substantial with Gibbs free energies of activation between 19 and 40 kcal mol^–1^. Competing reaction channels include Wittig olefination (for ketones and aldehydes), as well as Claisen condensation reactions. The reaction offers an alternative entry point to the nucleophile-catalyzed Cloke-Wilson rearrangement.

## Introduction

Schöllkopf and Wittig’s discovery of the olefination of ketones via reaction with triphenylphosphonium ylides^1,2^ opened the path to the discovery of an array of related reactions, involving organophosphorus^3,4^ as well as organotitanium compounds.^5,6^ The parent Wittig reaction involves concerted (yet asynchronous)^7^ formation of an oxaphosphetane ring, followed by cycloreversion to yield triphenylphosphine oxide and an alkene. Betaines are unlikely to be involved under normal reaction conditions.^8^ However, computational work suggests that in very polar solvents, betaines can be formed as well, and that entropy plays an important role in the reaction outcome.^9^ It works well for ketones and aldehydes but not for esters, lactones, amides, and lactams. The Wittig reaction has been investigated in a number of computational studies,^9−13^ and its stereochemical outcome (*E*- vs *Z*-olefin formation) is now well understood. Depending on the nature of the ylide involved (stabilized or nonstabilized), formation of the oxaphosphetane intermediate either goes through a very early puckered transition state structure leading to the *Z*-alkene (unstabilized ylides),^8,11,14,15^ or through a tighter transition state governed by dipolar interactions, resulting in *E*-alkenes (stabilized ylides). There appears to be a correlation between the degree of bond formation in the transition state structure and the stereochemical outcome.^16^

In the case of α,β-unsaturated carbonyl compounds, in addition to the typical Wittig reaction, a conjugate addition can take place, which results in the formation of zwitterions.^17^ These zwitterions can undergo synthetically useful follow-up reactions. Formation of cyclopropanes, via intramolecular S_N_2 (or S_N_*i*) reaction, has been observed and used in a few systems.^17−21^ It is noted, however, that this reaction has been neglected for a long time now and that there are only very few reports of its use in the literature and, so far, no computational study of it.

**Scheme 1:**
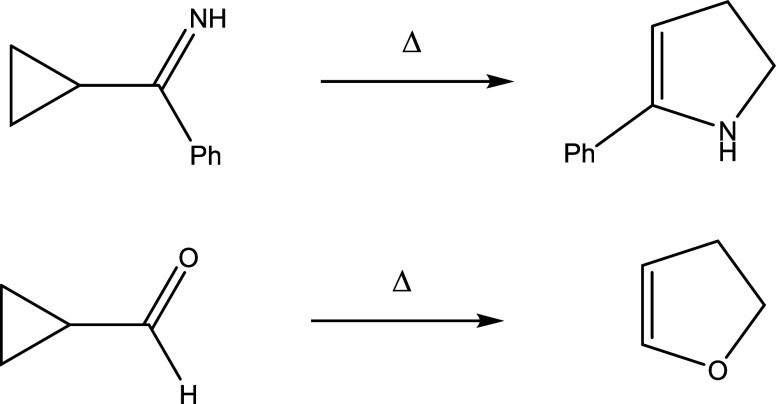
Original Examples for the Uncatalyzed Cloke-Wilson Rearrangement

Upon heating, vinylcyclopropane rearranges into cyclopentene in a process that, depending on the substitution, can either be described as a concerted 1,3-sigmatropic carbon shift, or as occurring via biradical intermediates.^20^ Similarly, acyl cyclopropanes or imines derived thereof can be rearranged into dihydrofurans or dihydropyrroles in a process that is now known as the Cloke-Wilson rearrangement, after the researchers who first described it^23,24^ (Scheme 1).

**Scheme 2:**
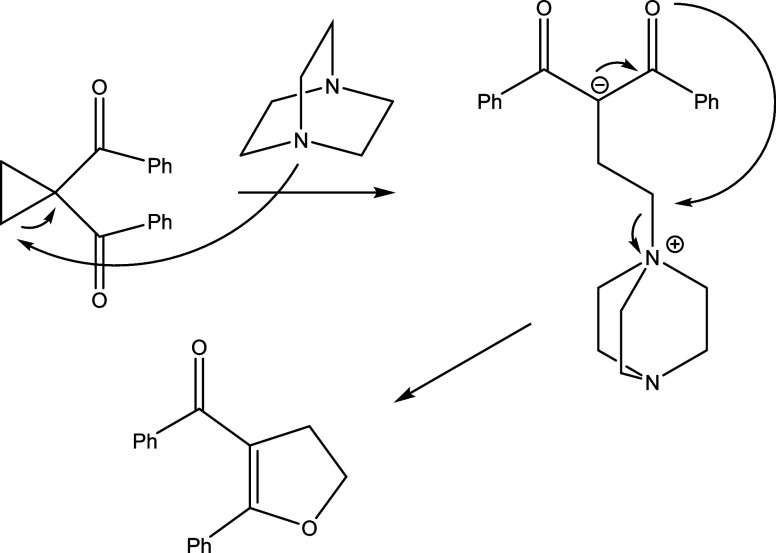
Mechanism of the DABCO-Catalyzed Cloke-Wilson Rearrangement^33^

While the uncatalyzed Cloke-Wilson rearrangement suffers from rather harsh reaction conditions required (180 °C for Cloke’s synthesis of dihydropyrroles and about 400 °C for Wilson’s rearrangement of cyclopropanecarbaldehyde), it can be catalyzed by transition metal complexes such as Ni(COD)_2_,^25^ photoredox catalysis,^26^ organocatalysis,^27^ electrophiles,^28^ acids,^29^ and nucleophiles.^30^ Among the latter, 1,4-diazabicyclo[2.2.2]octane (DABCO) has been used to catalyze the reaction.^31−33^ The reaction mechanism involves formation of zwitterionic species, as shown in Scheme 2.

**Scheme 3:**
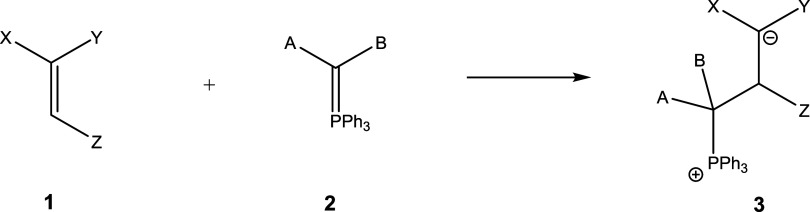
General Reaction Scheme for the Addition of Phosphoranes to Acceptor-Substituted Alkenes

**Scheme 4:**
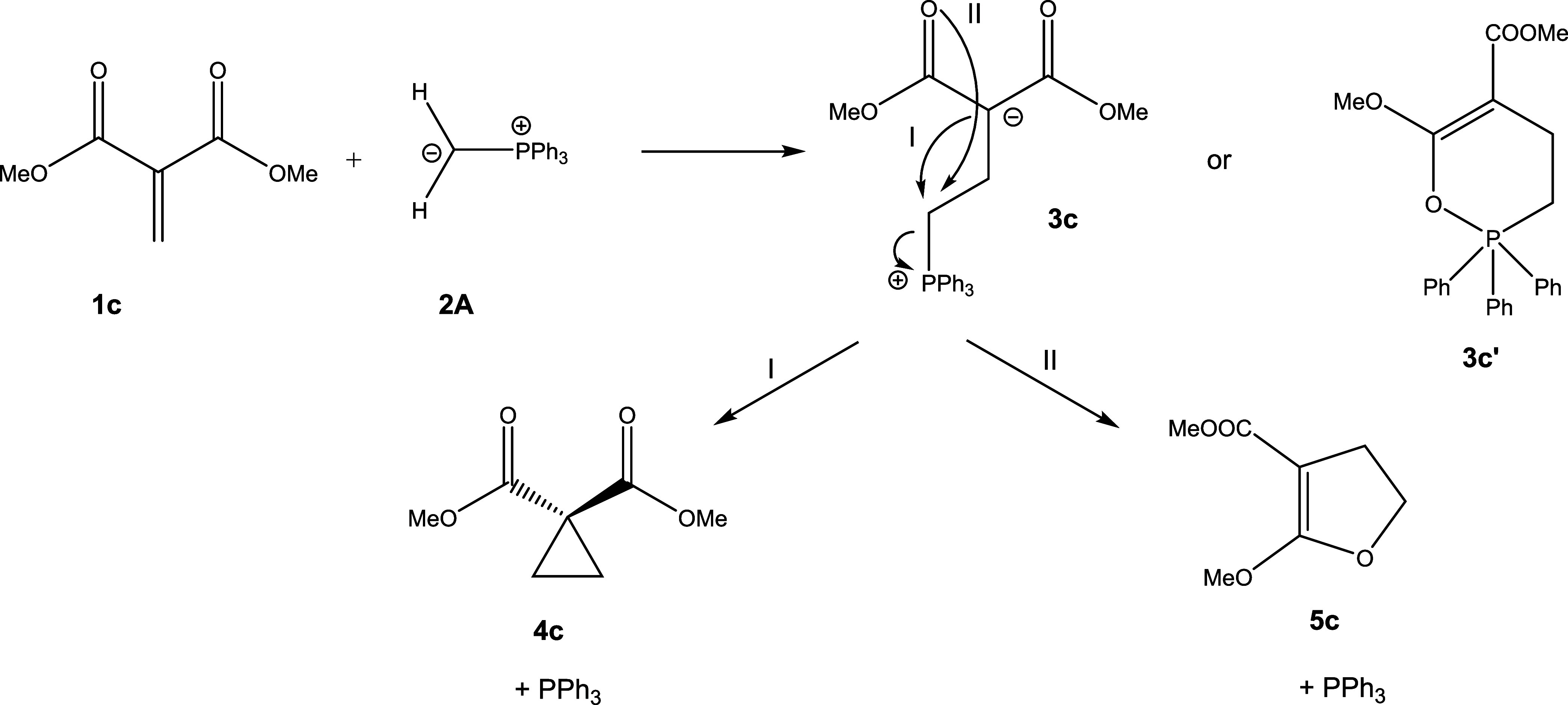
Reaction Channels Following Initial Formation of Zwitterion **3c**

This contribution looks at conjugate addition to the β-position of Michael acceptors (**1**), employing density functional theory as well as *ab initio* theory. Acceptor substituents investigated are ketone, nitrile, ester, amide, thioester, phosphonate ester, and nitro functionalities. The primarily formed products could be zwitterions (**3**) (formed via conjugate addition) or oxaphosphinines (**3′**) (formed via a formal 4π + 2π cycloaddition), which could be followed up by cyclopropane (**4**) or dihydrofuran (**5**) formation via intramolecular S_N_2 (or S_N_*i*) reactions. Schemes 3 and 4 illustrate the structures involved.

**Scheme 5:**
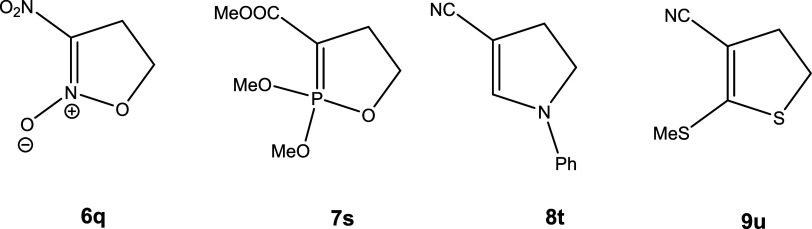
Examples of Other 5-Membered Ring Compounds Potentially Formed in Cyclizations of Derivatives of **3**

Other activating functionalities would introduce different nucleophilic centers into the system, thus generating the opportunity to yield cyclic nitrones **6** (X = NO_2_); phosphoranes **7** (X = PO(OMe)_2_); dihydropyrroles **8** (X = CH=NPh); and dihydrothiophenes **9** (X = CSSMe) (Scheme 5). In addition, the zwitterions **3** can also be in equilibrium with a six-membered ring oxaphospholene isomer **3′**. The systems calculated are shown in Schemes 1 (specific example) and 2, and the results are presented in Table 1. We studied the reactions of **1** with three different Wittig ylides, the methylene phosphorane **2A** (A = B = H), the ethylidene phosphorane **2B** (A = CH_3_, B = H), and the isopropylidene phosphorane **2C** (A = B = CH_3_).

**Table 1:** Gibbs Free Energies, Relative to the Sum of Energies of **1** + **2**, Calculated Using DLPNO–CCSD(T)/def2-TZVP(CPCM(C),THF)//M06-2X/cc-pVDZ(pcm,THF), with Thermal Correction from DFT

system	precursor **1**	phosphorane **2**	TS **1** + **2** → **3**	**3**	TS **3** → **4**^a^	**4**^a^	TS **3** → **5**^a^	**5**^a^	TS **3** → (**6**–**9**)^a^	**6**–**9**^a^
a	X = Y = COCH_3_, Z = H	A = B = H	16.1	–18.8	10.6	–21.2	13.2	–29.3		
b	X = COCH_3_, Y = Z = H	A = B = H	20.8	–1.9	21.4	–23.1	28.6	–21.8		
c	X = Y = COOCH_3_, Z = H	A = B = H	14.4	–15.2	12.6	–25.1	21.7	–14.5		
d	X = Y = COOCH_3_, Z = H	A = CH_3_, B = H	12.5	–18.4	7.4	–31.5	17.6	–23.1		
e	X = Y = COOCH_3_, Z = H	A = B = CH_3_	9.9	–17.2	6.1	–34.1	14.1	–30.6		
f	X = COOCH_3_, Y = Z = H	A = B = H	21.2	3.8	25.1	–22.0	38.5	–2.6		
g	X = Y = COOCH_3_, Z = Ph	A = B = H	17.8	–11.1	16.5	–23.9	22.5	–11.9		
h	X = Y = COOCH_3_, Z = Ph	A = B = CH_3_	15.7	–8.0	14.4	–31.0	17.3	–29.0		
i	X = Y = COOCH_3_, Z = 4-MeOPh	A = B = H	17.1	–10.6	15.9	–23.6	22.5	–11.6		
j	X = Y = COOCH_3_, Z = 4-MeOPh	A = B = CH_3_	15.5	–5.9	14.4	–30.5	17.4	–28.3		
k	X = Y = COOCH_3_, Z = 4-NO_2_Ph	A = B = H	13.7	–12.0	16.5	–24.8	19.9	–13.2		
l	X = Y = COOCH_3_, Z = 4-NO_2_Ph	A = B = CH_3_	13.6	–10.0	12.9	–32.1	14.6	–30.1		
m	X = Y = CON(CH_3_)_2_, Z = H	A = B = H	17.6	–3.1	20.4	–23.5	31.3	–11.9		
n	X = COOCH_3_, Y = CN, Z = H	A = B = H	8.8	–18.5	11.5	–24.0	22.2	–13.2		
o	X = Y = CN, Z = H	A = B = H	7.6	–21.3	12.2	–23.1				
p	X = CN, Y = Z = H	A = B = H	10.1	6.4	25.0	–22.0				
q	X = Y = NO_2_, Z = H	A = B = H	no barrier	–34.1	5.6	–27.2			4.7	–27.3
r	X = NO_2_, Y = Z = H	A = B = H	14.9	–13.6	17.7	–23.5			18.5	–24.3
s	X = COOCH_3_, Y = PO(OMe_2_), Z = H	A = B = H	12.5	–14.3	12.7	–26.3	25.3	–13.8	31.1	–2.3
t	X = (*E*) – CH = NPh, Y = CN, Z = H	A = B = H	12.7	–17.3	13.3	–21.0			16.2	–36.6
u	X = CSSCH_3_, Y = CN, Z = H	A = B = H	19.7	–26.3	9.1	–22.1			6.4	–39.2

a Plus triphenylphosphine.

It is noted that the structures of all stationary points calculated, if not already shown in a figure in this article, are found in the Supporting Information (Figures S1–S17).

## Results and Discussion

Due to the size of the molecules investigated, geometry optimizations and frequency calculations (which would be required for calculation of enthalpies, entropies, and Gibbs free energies) at the DLPNO–CCSD(T) level of theory would not have been feasible. To calculate Gibbs free energies based on the results of the coupled cluster single point energy calculations, a hybrid approach was used. The Gibbs free energies listed were calculated by adding the “Thermal correction to Gibbs free energy”, as obtained in the DFT frequency calculations performed using Gaussian, to the DLPNO–CCSD(T) electronic energies of the stationary points optimized. The values refer to standard conditions (*T* = 298 K). The reference points of the calculations are the sums of the electronic energies of carbonyl compounds **1** and phosphoranes **2**. In one case, the reaction of the extremely electrophilic 1,1-dinitroethene (system **q**), a transition state structure could not be localized using DFT, and the reaction presumably proceeds barrierless. The results of the calculations are shown in Table 1 (Gibbs free energies), Table S1 (see the Supporting Information, selected calculated geometric parameters), and Table S2 (see the Supporting Information, DLPNO–CCSD(T) electronic energies and Gibbs free energies calculated purely by DFT).

The initial step, addition of the phosphoranes to the Michael acceptors, reduces the number of translational degrees of freedom available to the system and is, therefore, entropically unfavorable. This is reflected in the fact that both the Gibbs free energies of activation for addition of the phosphoranes to the Michael acceptors and the Gibbs free energies of formation of the zwitterions **3** are consistently less favorable by ca. 10–15 kcal mol^–1^ than the corresponding electronic energies of activation and reaction.

In this work, the focus is on the conjugate addition of the phosphorane. Conventional Wittig olefination, however, still needs to be considered, particularly for the ketones studied (systems **a** and **b**).

### Ketones

**Figure 1:**
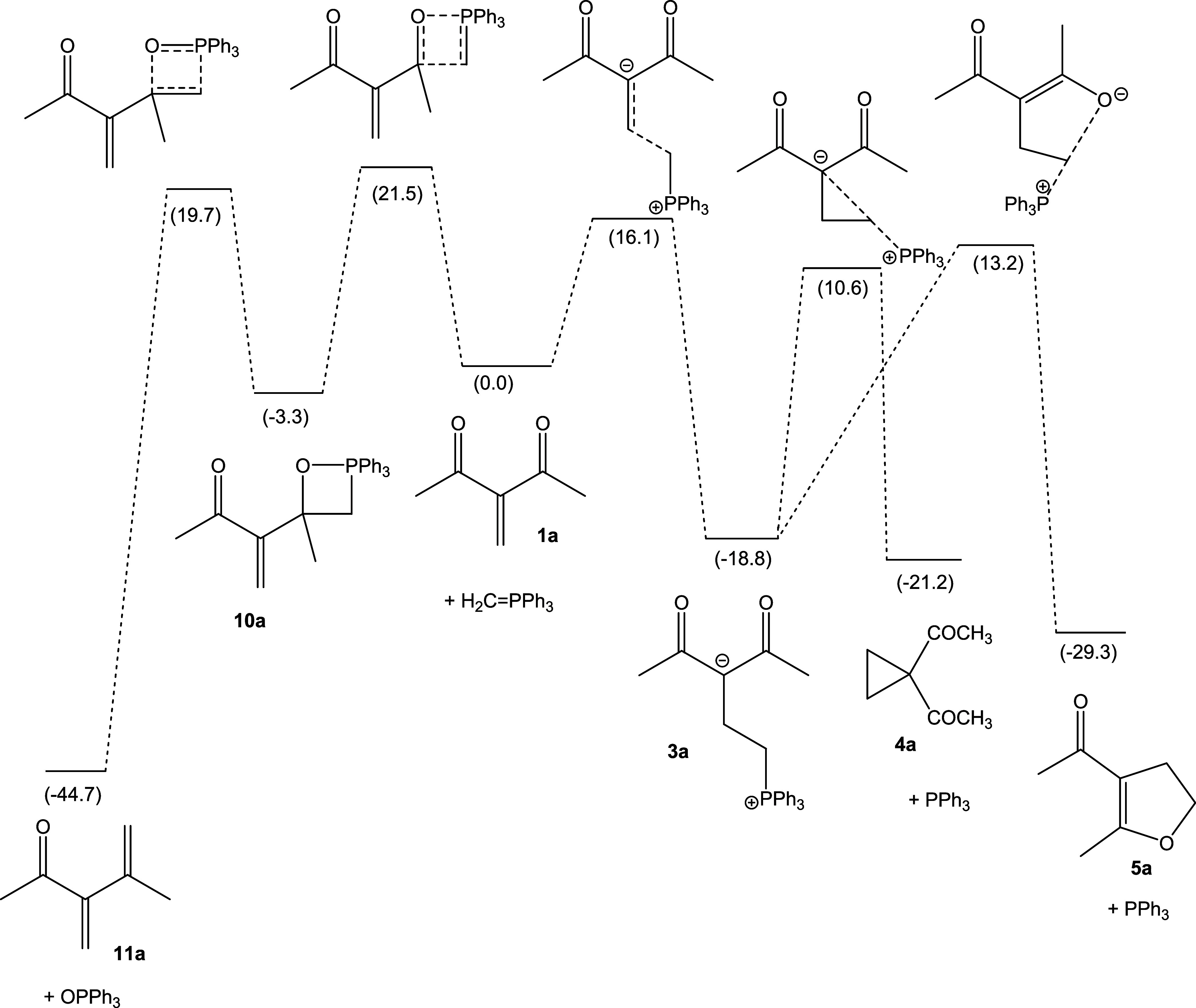
Schematic representation of the PES for the reaction of **1a** with triphenylphosphonium methylide. Gibbs free energies were based on DLPNO–CCSD(T)/def2-TZVP(CPCM(C),THF)//M06-2X/cc-pVDZ(THF) electronic energies, with thermal correction from M06-2X/cc-pVDZ(THF), in kcal mol^–1^, relative to **1a** + CH_2_=PPh_3_.

For methylene acetylacetone (**a**), the Gibbs free energy of activation for oxaphosphetane (**10a**) formation is calculated (DLPNO–CCSD(T)/def2-TZVP(CPCM(C),THF)//M06-2X/cc-pVDZ(THF)) as Δ*G*^⧧^ = 21.5 kcal mol^–1^. Formation of **10a** is calculated to be exergonic by Δ*G* = −3.3 kcal mol^–1^. The final cycloreversion step, with the formation of dienone **11a** and triphenylphosphine oxide, is calculated to be exergonic (relative to the starting materials **1a** and **2**) by Δ*G* = −44.7 kcal mol^–1^, and it is impeded by a Gibbs free energy of activation of Δ*G*^⧧^ = 23.0 kcal mol^–1^ (relative to **10a**). The barrier for the formation of zwitterion **3a** is calculated to be smaller than the barrier for Wittig olefination (Δ*G*^⧧^ = 16.1 kcal mol^–1^). While zwitterion **3a** is calculated to be significantly lower in energy than oxaphosphetane **10a**, the products of the Wittig reaction, diene **11a** and triphenylphosphine oxide, are the global minimum on the potential energy hypersurface (PES). Hence, under thermodynamic reaction control, the reaction of **1a** with the parent triphenylphosphonium methylide should preferentially yield the Wittig-type product **11a**, but the kinetic preference is for the reaction channel leading to zwitterion **3a** and further to cyclopropane **4a**. On the conjugate addition side of the PES, dihydrofuran **5a** is calculated to be lower in energy than cyclopropane **4a**, in agreement with experimental results that demonstrate the feasibility of the Cloke-Wilson rearrangement of diacyl cyclopropanes. The results of the reactions of **1a** are shown in Figure 1.

In the case of methylvinylketone as electrophile (system **b**), the findings differ from system **a** in that both the thermodynamic and kinetic preferences are for Wittig olefination, see Figure S1 (Supporting Information). On the conjugate addition side, the cyclopropane is now more favored, both thermodynamically and kinetically.

### Esters

**Figure 2:**
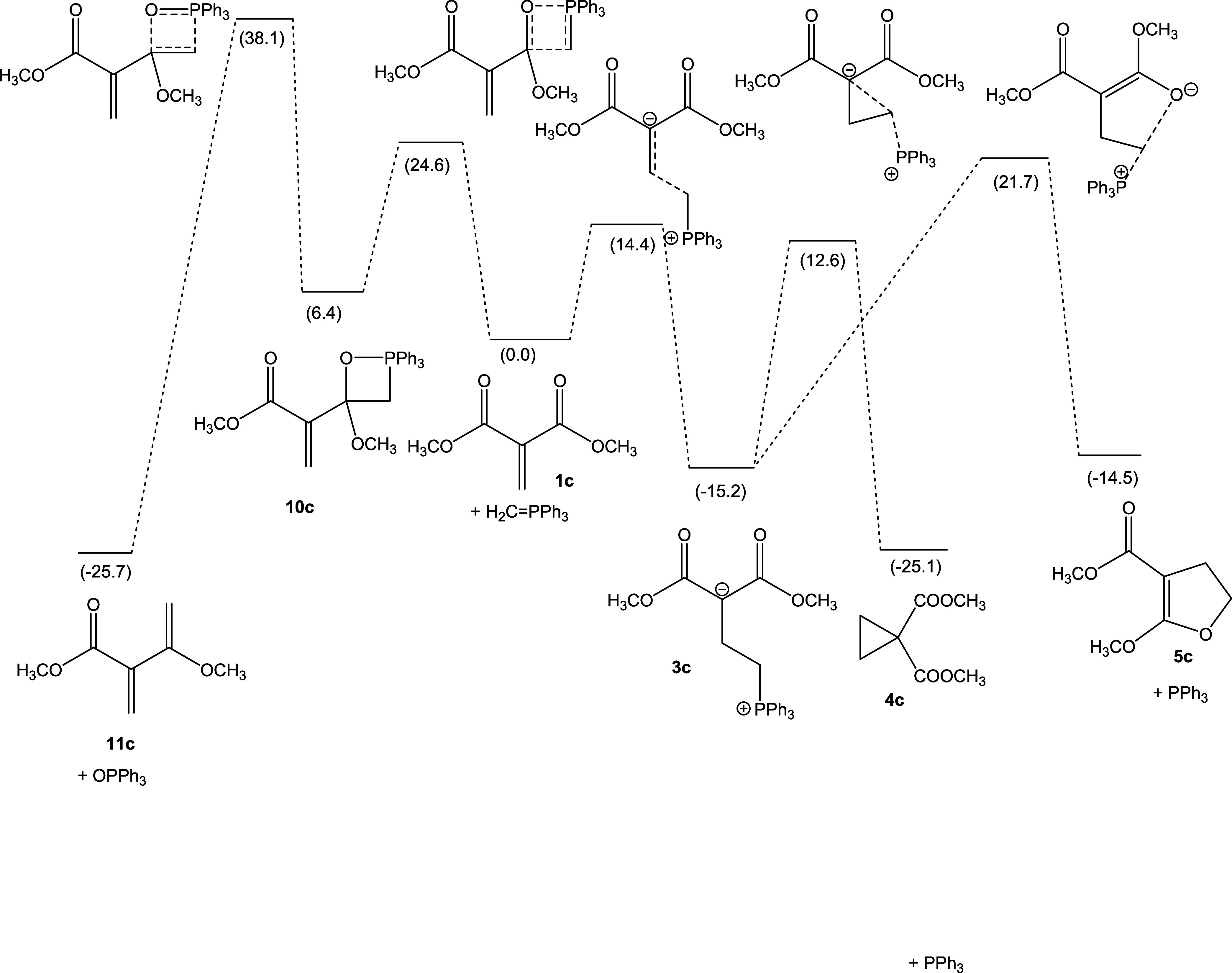
Schematic representation of the PES for the reaction of **1c** with triphenylphosphonium methylide. Gibbs free energies are based on DLPNO–CCSD(T)/def2-TZVP(CPCM(C),THF)//M06-2X/cc-pVDZ(THF) electronic energies, with thermal correction from M06-2X/cc-pVDZ(THF), in kcal mol^–1^, relative to **1c** + CH_2_=PPh_3_.

If the ketone moieties are replaced by ester groups, as in dimethylmethylenemalonate **1c**, then the selectivity of the reaction is changed, now preferentially yielding the cyclopropane product **4c**, both kinetically and thermodynamically. Figure 2 shows the results.

Figure 2 shows that the formation of zwitterion **3c** should be a fast process for **1c**. However, the follow-up cyclization of **3c** to cyclopropane **4c**, while thermodynamically favorable, is predicted to be impeded by a significant barrier of Δ*G*^⧧^ = 27.8 kcal mol^–1^. This places the reaction lifetime for the formation of **4c** in the range of ca. 3 h, even at elevated temperatures (*T* = 400 K).^34^ Under these circumstances, side reactions such as the Claisen condensation might become competitive. If the ethylidene phosphorane **2B** or the isopropylidene phosphorane **2C** is reacted with **1c**, the barriers of both the conjugate addition and the Wittig reaction go down with increasing degree of alkylation of the phosphorane, but the conjugate addition should still be the favored reaction pathway (Figures S2 and S3, see Supporting Information). Remarkably, the barriers for formation of both cyclopropane **4e** and dihydrofuran **5e** from zwitterion **3e** (see Figure S3, Supporting Information) are calculated to be somewhat smaller than the corresponding barriers in system **3c**, which lacks the methyl groups. Cyclopropane **4c** is calculated to be lower in energy than dihydrofuran **5c**, and likewise, **4d** and **4e** are lower in energy than **5d** and **5e**, respectively, indicating that for the esters, the equilibrium of Cloke-Wilson rearrangement should lie on the side of the cyclopropanes.

**Figure 3:**
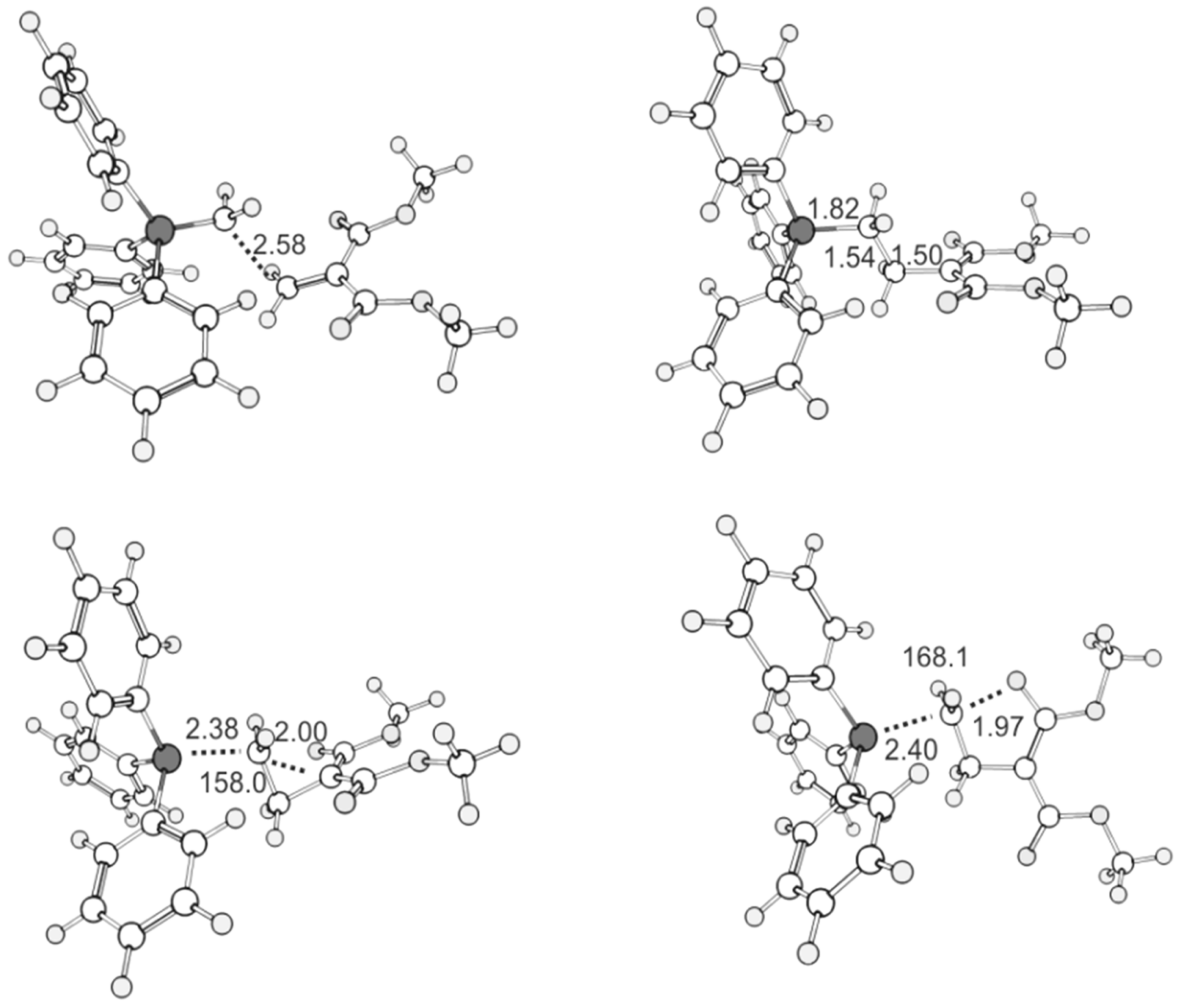
Stationary points in system **c**, as optimized at the M06-2X/cc-pVDZ(THF) level of theory. Top left: transition state structure for the formation of **3c**. Top right: zwitterion **3c**. Bottom left: transition state structure for the formation of cyclopropane **4c** + PPh_3_. Bottom right: transition state structure for the formation of dihydrofuran **5c** + PPh_3_. Selected bond atomic distances in Å, angles in °. Dark gray large circles: phosphorus; light gray medium size circles: oxygen; white medium size circles: carbon, light gray small circles: hydrogen.

**Figure 4:**
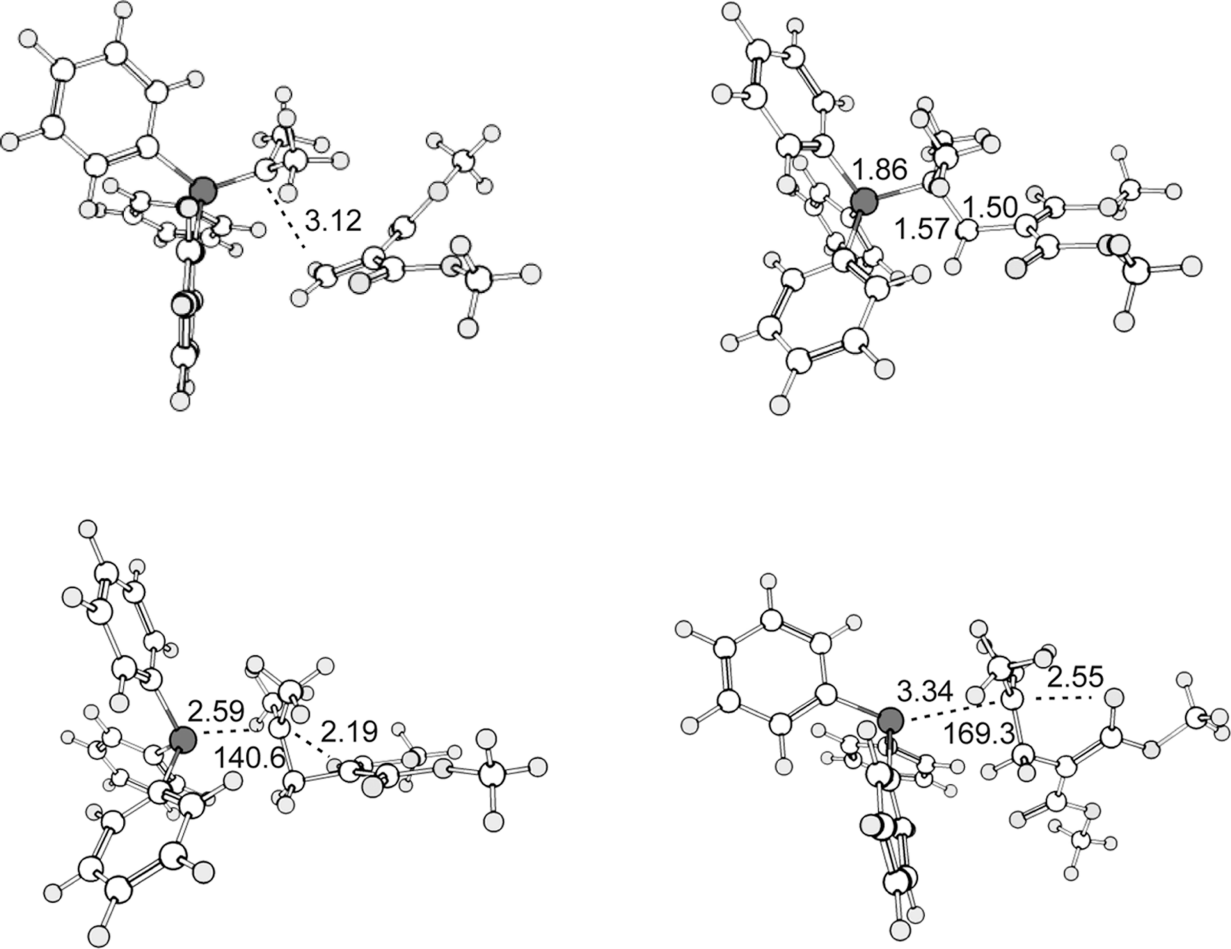
Stationary points in the system **e**, as optimized at the M06-2X/cc-pVDZ(THF) level of theory. Top left: transition state structure for the formation of **3e**. Top right: zwitterion **3e**. Bottom left: transition state structure for the formation of cyclopropane **4e** + PPh_3_. Bottom right: transition state structure for the formation of dihydrofuran **5e** + PPh_3_. Selected bond atomic distances in Å and angles in °. Dark gray large circles: phosphorus; light gray medium size circles: oxygen; white medium size circles: carbon, light gray small circles: hydrogen.

Figure 3 shows the optimized structures on the hypersurface of the reaction of dimethylmethylenemalonate **1c** and the parent Wittig ylide **2A** (system c), and Figure 4 shows the corresponding stationary points in the reaction of **1c** and isopropylidene phosphorane **2C** (system **e**). The transition state geometries for the initial conjugate addition step feature a long C–C distance of R_C–C_ = 2.58 Å (system **c**) or even 3.12 Å (system **e**). Hence, while the steric effect of the additional methyl groups does come into play at the TS geometry, it is a very early transition state structure anyway, and the increased nucleophilicity of the ethylidene or isopropylidene phosphorane is important. The follow-up reactions involved in the decay of the zwitterions **3** are intramolecular S_N_2 (i.e., S_N_*i*) reactions, as is clearly seen from the transition state structures, see Figures 1 and 2, bottom.

Going to the sterically more demanding isopropylidene phosphorane, the C–C and C–P distances in the reaction coordinates of all three transition state structures investigated become significantly longer, see Figure 4. The barriers, however, are not increased, but—surprisingly—are lower (Δ*G*^⧧^ for formation of **4c**: 27.8 kcal mol^–1^, Δ*G*^⧧^ for formation of **5c**: 36.9 kcal mol^–1^; Δ*G*^⧧^ for formation of **4e**: 23.3 kcal mol^–1^, Δ*G*^⧧^ for formation of **5e**: 31.3 kcal mol^–1^), see also Figure S3.

The reactivity predicted for methyl acrylate **1f** parallels the reactivity calculated for **1c**, in that the barriers in the conjugate addition pathway are smaller than the barriers for Wittig olefination. Overall, the conjugate addition pathway is less exergonic here than that for **1c** and has a higher barrier. This resembles the situation found for **1a** vs **1b**. Introducing a cyano group as second activating substituent, as in **1n**, has the effect of reducing the barrier for conjugate addition again, see Figure S12. System **f** was the only system for which an oxaphospholene isomer (**3f’**) to a zwitterion (**3f**) could be localized by DFT, see Figure S4, SI. Cyclic structure **3f’** is calculated to be slightly higher in free energy than **3f**, while being slightly more favorable in electronic energy. This finding makes sense in that among the carbonyl-based zwitterions **3**, **3f** is the one in which the negative charge is least stabilized and the cyclic structure offering less rotational degrees of freedom is entropically disfavored.

The presence of an additional benzene ring, as in systems **g**-**l**, slightly increases the free energy of activation for initial zwitterion formation and makes the reaction less exergonic. Again, the isopropylidene phosphoranes (systems **h**, **j**, and **l**) are predicted to react faster than the methylidene phosphoranes (systems **g**, **i**, and **k**). Among the three aromatic Michael acceptors studied, the *p*-nitro-substituted system **k**,**l** is predicted to be the most reactive, in agreement with the expectation that the position α to the benzene ring should be more electrophilic if a *p*-nitro functionality is present (see Figures S5–S10). Again, barriers for the conjugate addition reactions are predicted to be smaller for the sterically more demanding isopropylidene phosphorane **2C**.

### Other Functionalities: Amide, Cyano, Nitro, Imine, Thioester

#### Amides

The methylenemalonic diamide (system **m**) is predicted to be rather unreactive (see Figure S11), in agreement with the fact that amide groups are less electron-withdrawing than ester, nitrile, or nitro functionalities. Conjugate addition should preferentially yield cyclopropane **4m**.

#### Phosphonates

The mixed ester/phosphonate ester system **s** (see Figure S17) is predicted to preferentially result in cyclopropanation, as both the formation of the dihydrofuran **5s** and formation of the cyclic phosphorane **7s** are calculated to be less favorable.

#### Nitriles

In the case of the nitriles **1o** and **1p**, it is again the doubly activated methylenemalodinitrile **1o** that should show a far larger reactivity compared to acrylonitrile **1p**. In the former, formation of cyclopropane **4o** and triphenylphosphine is calculated to be significantly endothermic (yet exergonic, due to the entropically favorable release of triphenylphosphine) relative to zwitterion **3o**, whereas the situation is different for zwitterion **3p**, where formation of cyanocyclopropane **4p** is calculated to be both exothermic and exergonic, see Figures S13 and S14 and Tables S1 and S2.

#### Nitroalkenes

1,1-Dinitroethylene **1q** is an extremely electrophilic Michael acceptor that is predicted to react with parent phosphorane **2A** in a barrierless and strongly exergonic (Δ*G* = −34.1 kcal mol^–1^) reaction. Follow-up reactions, to either 1,1-dinitrocyclopropane **4q** or the cyclic nitrone **6q**, are significantly endergonic from **4q** and unlikely to be practicable, see Figure S15. The situation is different for nitroethene **1r**, where the formation of nitrocyclopropane **4r** should be the kinetic reaction outcome, whereas the cyclic nitrone **6r** should be the product under thermodynamic reaction control, see Figure S16. It is noted, though, that the differences between the two reaction channels in terms of both free energies of activation and free energies are very small for system **r**.

#### Imines

**Figure 5:**
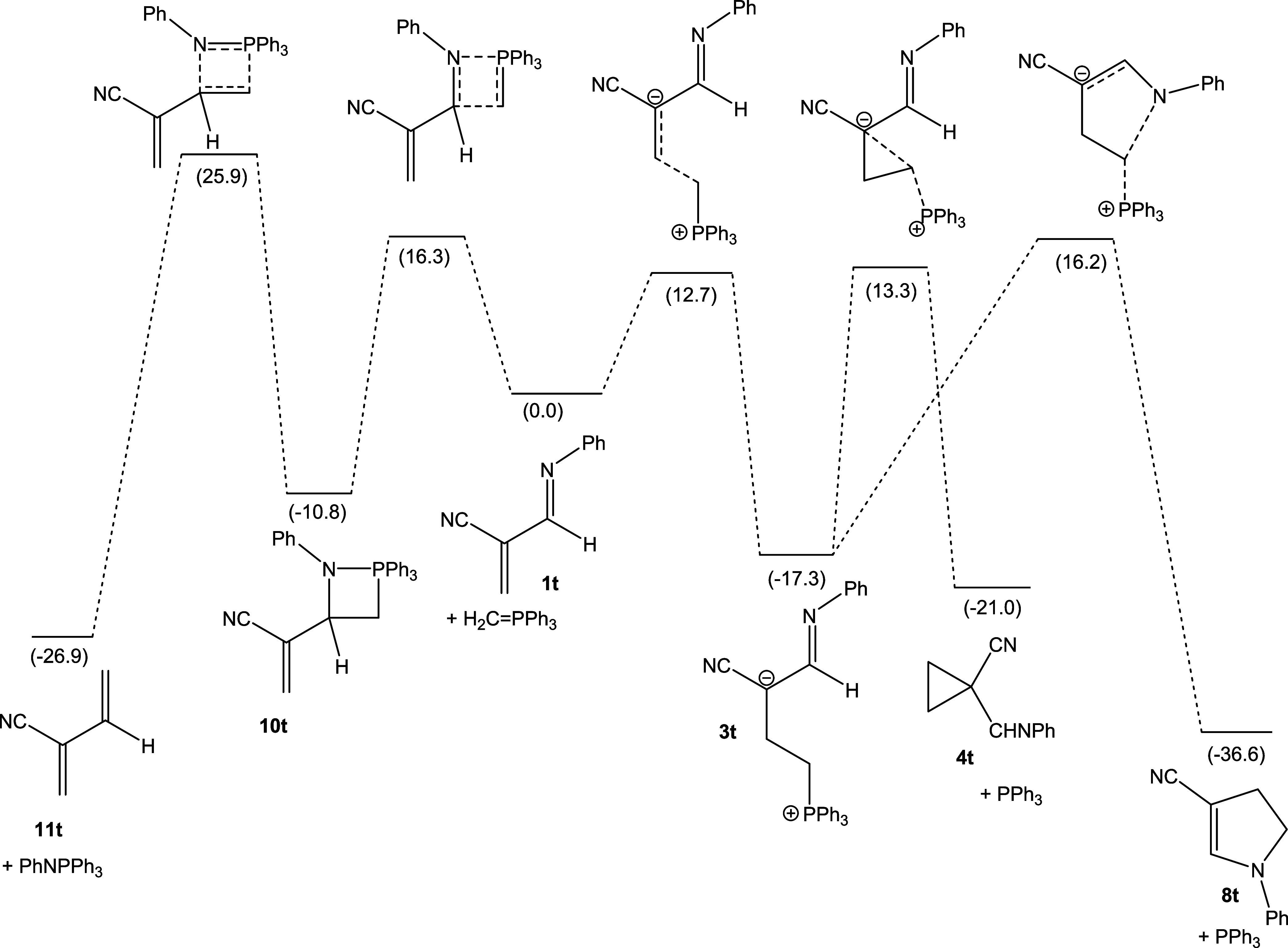
Schematic representation of the PES for the reaction of **1t** with triphenylphosphonium methylide. Gibbs free energies are based on DLPNO–CCSD(T)/def2-TZVP(CPCM(C),THF)//M06-2X/cc-pVDZ(THF) electronic energies, with thermal correction from M06-2X/cc-pVDZ(THF) in kcal mol^–1^, relative to **1t** + CH_2_=PPh_3_.

Of interest is the cyano-substituted *N*-phenylimine (system **t**), where the formation of a dihydropyrrole (**5t**) is calculated to be the thermodynamically most favorable reaction outcome (Figure 5), while both an alternative aza-Wittig reaction and formation of cyclopropane **4t** are predicted to be less exergonic. Hence, this system holds promise as a potential access to dihydropyrrole derivatives, akin to Cloke’s original imine rearrangement.^21,22^

#### Thioesters

**Figure 6:**
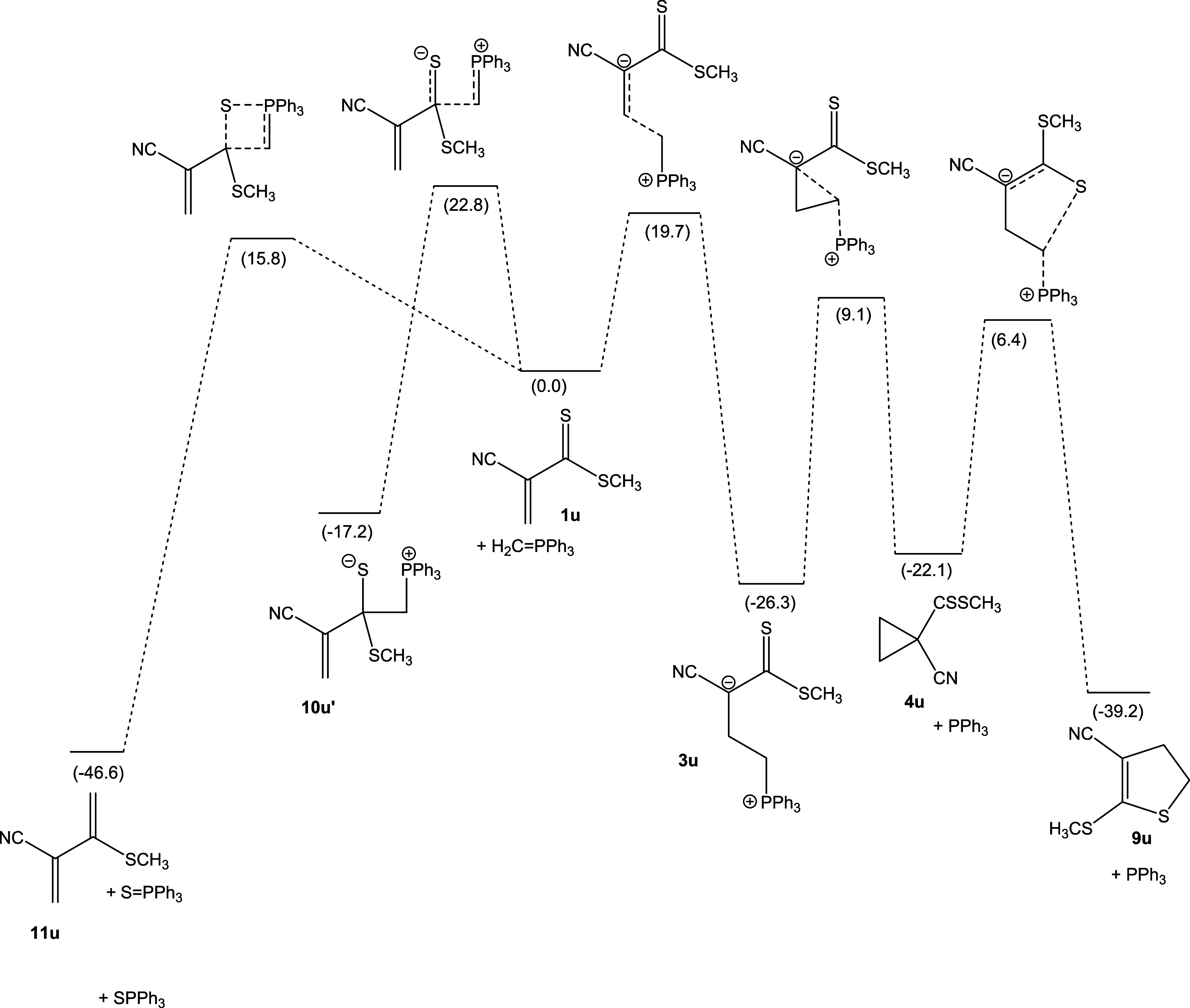
Schematic representation of the PES for the reaction of **1u** with triphenylphosphonium methylide. Gibbs free energies were based on DLPNO–CCSD(T)/def2-TZVP(CPCM(C),THF)//M06-2X/cc-pVDZ(THF) electronic energies, with thermal correction from M06-2X/cc-pVDZ(THF) in kcal mol^–1^, relative to **1u** + CH_2_=PPh_3_.

In the case of the thioester system **u**, the higher reactivity of the C=S bond compared to a C=O bond results in the thia-Wittig reaction being predicted to have a fairly small barrier. The TS for the concerted 2 + 2 cycloaddition–cycloreversion sequence (the thiaphosphetane **10u** is not predicted to be a minimum structure) is predicted to be higher in Gibbs free energy by 15.8 kcal mol^–1^. A betaine structure **10u’** is identified as a stationary point in at least one rotameric form but likely does not play a role in the chemistry, as its formation from the precursors is predicted to be slower than the concerted cycloaddition-cycloreversion reaction. Formation of zwitterion **3u** is predicted to be facile and exergonic but slower than the thia-Wittig reaction. Among the two follow-up cyclization reactions, the formation of dihydrothiophene **5u** is predicted to be favored, both thermodynamically and kinetically. This is likely due to the increased nucleophilicity of the thiocarbonyl sulfur atom, as compared to a carbonyl oxygen in other systems. Figure 6 shows the electronic energies of a range of calculated stationary points.

**Scheme 6:**
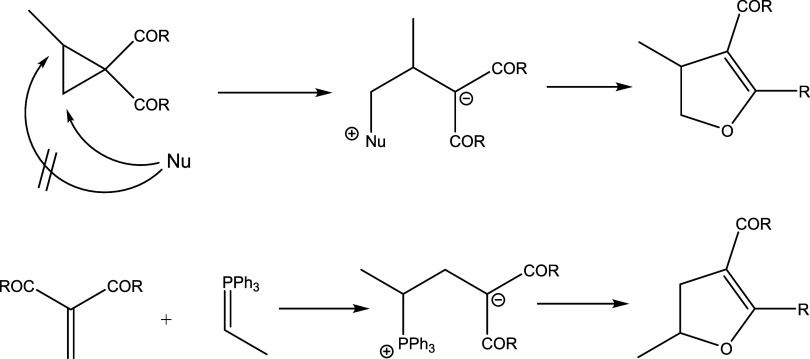
Example for Different Regiochemical Outcomes of the Cloke-Wilson Rearrangement If Accessed from the Cyclopropane (Top) or the Phosphorane (Bottom)

The results presented so far indicate that conjugate addition of (unstabilized) phosphoranes to acceptor-substituted alkenes in most cases will be a facile process, yielding zwitterionic species **3** in reactions that in most cases are significantly exergonic. Exceptions are alkenes activated by only one electron-withdrawing group such as acrylonitrile **1p**. Follow-up reactions involve ring closure to cyclopropanes and to 5-ring heterocycles such as dihydrofurans or dihydropyrroles. The barriers calculated for the follow-up reactions, however, in most cases are significant, and the reactions will be slow. This implies that intermolecular reactions, which were not investigated here, might become competitive. In addition, at least for unsaturated ketones, and certainly also aldehydes, conventional Wittig olefination is predicted to compete efficiently with the conjugate addition. Does this imply that the reaction is confined to remain a laboratory curiosity? This does not necessarily have to be the case. It is important to realize that a barrier of 30 kcal mol^–1^ for the formation of a secondary product from a zwitterion **3** at a temperature *T* = 125 °C translates into a reaction duration of slightly more than an hour.^34^ It is also noted that the reaction provides an additional entry point to the nucleophile-catalyzed Cloke-Wilson rearrangement of cyclopropyl ketones to dihydrofurans.^32^ The latter is important if the cyclopropane precursor required for this reaction is not easily accessible. Using the phosphorane to access the zwitterion, it should also in principle be possible to access a different regioisomer of the dihydrofuran product, as shown exemplarily in Scheme 6. Starting from the cyclopropane, the nucleophile will invariably attack the sterically less encumbered carbon atom of the cyclopropane moiety, whereas the other regioisomer of the zwitterion necessarily is formed by conjugate addition of the phosphorane. Very likely, however, this approach at regiocontrol should fail for systems in which the cyclopropane is the product formed under kinetic reaction control (i.e., most of the systems studied here except for the thiocarbonyl system **u**).

One result from this work requires commenting on. Comparing the reaction of dimethylmethylenemalonate **1c** with the parent phosphorane **2A** and the isopropylidene phosphorane **2C**, we find that the barrier for formation of zwitterion **3c** (from **1c** and **2A**, Δ*G*^⧧^ = 14.4 kcal mol^–1^ relative to the free reactants) is larger than the barrier of formation of zwitterion **3e** (from **1c** and **2C**, Δ*G*^⧧^ = 9.9 kcal mol^–1^ relative to the free reactants). While this is likely due to a very early transition state structure in which steric effects do not strongly come into play, the prediction that the barrier for both the S_N_i reactions yielding the cyclopropane **4** and the dihydrofuran **5** is smaller for system **e** (Δ*G*^⧧^ for the formation of **4e**: 23.3 kcal mol^–1^, Δ*G*^⧧^ for the formation of **5e**: 31.3 kcal mol^–1^) than for the less sterically demanding system **c** (Δ*G*^⧧^ for the formation of **4c**: 27.8 kcal mol^–1^, Δ*G*^⧧^ for the formation of **5c**: 36.9 kcal mol^–1^) is remarkable. After all, it is generally thought (and taught) that S_N_2 and S_N_*i* reactions work best for reactions on primary substrates (here: a primary phosphonium cation as in **3c**), worse for secondary substrates (as in **3d**), and not at all on tertiary substrates, as in **3e**. The fact that the formation of **4e** or **5e** are sterically highly demanding reactions is revealed by the significantly increased interatomic distances along the reaction coordinate in the TS, see Table S1 and Figures 3 and 4. In the TS leading to the formation of **5e** in particular, the C–P bond is stretched considerably, indicating that this intramolecular S_N_2-type reaction, while concerted, is not synchronous, with a carbocation-like geometry first being created on the reacting tertiary center, followed by bond formation with the incoming carbonyl oxygen atom. In the literature, precedent exists in the intramolecular reductive ring opening reaction of epoxide alcohols employing PhSiH_3_ as reductant, where an intramolecular S_N_2 reaction was also reported to occur preferentially on a tertiary carbon center rather than a secondary carbon center.^35^

**Figure 7:**
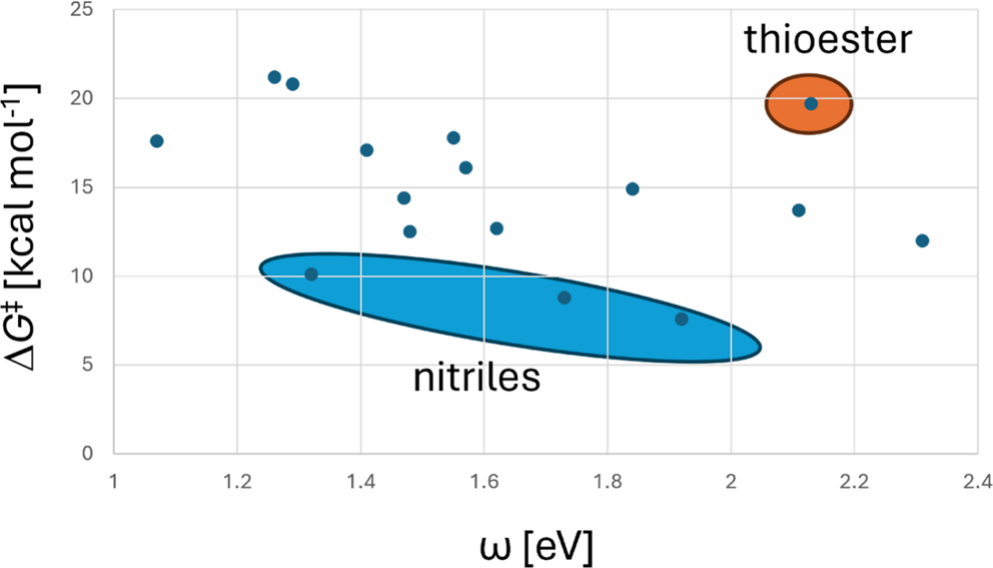
Plot of the Gibbs free energy of activation for the addition of parent Wittig ylide **2A** to acceptor-substituted alkenes **1** vs CDFT electrophilicity index parameter.

The Gibbs free energy of activation for the initial conjugate addition step is a function of the electrophilicity of the acceptor-substituted alkene. Based on the M06-2X/cc-pVDZ optimized geometries of the starting materials alkenes **1**, the energies of the HOMO and LUMO were evaluated for each. The framework of conceptual density functional theory (CDFT) defines a number of parameters (electronic chemical potential μ, hardness η, electrophilicity index ω,^36−38^ nucleophilicity index *N*(7,39)). The values calculated (see Table S3) confirm that the alkenes do indeed react as electrophiles, whereas the phosphoranes function as nucleophiles. 1,1-Dinitroethene **1q** in fact is calculated to be a weaker nucleophile (negative *N*) than tetracyanoethene used as reference molecule in the definition of *N*.^39^ Plotting the calculated Gibbs free energy of activation for the conjugate addition steps (reaction with ylide **2A**) versus the electrophilicity index ω (in eV) of the alkene (see Table S3), obtained from the highest occupied molecular orbital (HOMO) and least unoccupied molecular orbital (LUMO) energies, a somewhat noisy linear plot is obtained, Figure 7. The values for nitriles **1n**, **1o**, **1p** as well as the value for thioester **1u** are outliers (cyano-functionalized alkenes are thus predicted to show a particularly pronounced reactivity toward phosphoranes), whereas the carbonyl- (and nitro-, phosphonyl- and imino-) substituted alkenes are reasonably linear with ω. Ignoring the outliers, the slope obtained is  = −6.3 kcal mol^–1^ eV^–1^, with the outliers included  = −4.1 kcal mol^–1^ eV^–1^. The scatter seen in Figure 7 does not come as a surprise for several reasons. First, the electrophilicity parameter ω is a global parameter (derived from HOMO and LUMO energies) and therefore does not reflect the fact that attack of the phosphorane occurs locally at the β-carbon. Second, the effects of steric destabilization and stabilization via π-complex formation are ignored. Third, the Gibbs free energies of activation contain the entropy term, which does not contribute to ω at all.

## Conclusions

Reaction of unstabilized Wittig ylides with α,β-unsaturated carbonyl compounds and other acceptor-substituted alkenes is predicted to result in the formation of zwitterions by conjugate addition to the β-carbon atom of the alkene. The exceptions to this rule are α,β-unsubstituted ketones (and presumably also α,β-unsubstituted aldehydes, which were not investigated here), which preferentially react via conventional Wittig olefination, and thioester derivatives, where a thia-Wittig reaction is predicted to be prevalent. In the case of the most electron-deficient alkenes, the zwitterions formed are the lowest energy points along the reaction coordinate. In most other systems, the formation of cyclopropanes is predicted to be the most favorable reaction and also the kinetically most preferred outcome. Synthetically interesting other products, such as dihydrofurans or dihydrothiophenes, in most cases, are calculated to be disfavored, both kinetically and thermodynamically. A potential exception to this rule are α,β-unsaturated Schiff bases, where the formation of dihydropyrroles was calculated to be the thermodynamically most favorable outcome, in system **t**. The reaction may offer an alternative entry point to the nucleophile-catalyzed Cloke-Wilson rearrangement that could result in a different regiochemical outcome compared to the conventional reactions starting from a cyclopropane.

## Experimental Section and Computational Methods

All geometry optimizations and frequency calculations were performed employing the *Gaussian09* suite of programmes.^40^ All stationary points were fully optimized at the M06-2X/cc-pVDZ level of theory and characterized as minimum structures or transition state structures by performing a vibrational analysis.^41−44^ For all transition state structures along the reaction coordinates, for at least one system, an IRC calculation was performed to verify the nature of the reaction end points. The influence of solvation by THF was accounted for by using a polarizable continuum model (scrf = pcm,solvent = tetrahydrofuran).^45,46^ Single point energy calculations were performed at the DLPNO–CCSD(T)/def2-TZVP(CPCM(C),THF)//M06-2X/cc-pVDZ(THF) level of theory, employing the Domain Based Local Pair Natural Orbital method^47−49^ in combination with the CPCM (continuum solvation model with the conductor-like polarizable continuum model) method for approximation of the influence of solvation^50^ and Ahlrich’s def2-TZVP basis set.^51,52^ The older (T) rather than the more recent (T1) triple version was employed. These single point energy calculations were performed employing ORCA version 4.2.1.^53,54^ The CPCM model was used in combination with the COSMO epsilon function.^55^

## Data Availability

The data underlying this study are available in the published article and its Supporting Information.

## Supporting Information Available

Calculated energies of stationary points optimized (Tables S1–S3) (Figures S1–S17) (PDF)
Optimized geometries (XYZ)

## Supplementary Material

jo4c00757_si_001.pdf

jo4c00757_si_002.xyz
