# Supplementary material for: Accessing the Cloke-Wilson Rearrangement via Conjugate Addition of Phosphoranes to Michael Acceptors: A Route to Cyclopropanes and 5-Membered Ring Heterocycles Investigated by Density Functional and Ab Initio Theory
Source: J Org Chem. 2024 Aug 13;89(17):12020–31. doi: 10.1021/acs.joc.4c00757 (PMC11382343; doi:10.1021/acs.joc.4c00757)
Supplement: Supplementary file 1 — jo4c00757_si_001.pdf [file jo4c00757_si_001.pdf]

Accessing the Cloke-Wilson Rearrangement *via* Conjugate Addition of Phosphoranes to Michael Acceptors: A Route to Cyclopropanes and 5-Membered Ring Heterocycles Investigated by Density Functional and *Ab Initio* Theory.

Götz Bucher*

School of Chemistry, University of Glasgow, Joseph Black Building, University Avenue, Glasgow G12 8QQ, United Kingdom. goetz.bucher@glasgow.ac.uk

Supporting Information

Table of Contents:

Table S1 S2

Table S2 S3

Table S3 S5

Figure S1 S6

Figure S2 S7

Figure S3 S8

Figure S4 S9

Figure S5 S10

Figure S6 S11

Figure S7 S12

Figure S8 S13

Figure S9 S14

Figure S10 S15

Figure S11, Figure S12 S16

Figure S13, Figure S14 S17

Figure S15, Figure S16 S18

Figure S17 S19

Calculated energies S19

Table S1: Geometric parameters of transition state structures in the reactions investigated, systems **a**-**u**. (M06-2X/cc-pVDZ(THF)). Distances in Å, angles in °.

	Precursor	Phosphorane	R(C-C)<br>TS **1**+**2**→**3**	α<br>(C-C-C)[a]<br>TS **1**+**2**→**3**	R(C-C)<br>*R(C-P)*<br>TS **3**→**4**	α<br>(C-C-P)[b]<br>TS **3**→**4**	R (O-C)<br>*R(C-P)*<br>TS **3**→**5**	α<br>(O-C-P)[c]<br>TS **3**→**5**	R(X-C)<br>*R(C-P)*<br>TS **3**→(**6**-**9**)	α<br>(X-C-P)[d]<br>TS **3**→(**6**-**9**)
**a**	X = Y = $COCH_3$, Z = H	A = B = H	2.67	108.6	2.00<br>*2.39*	153.0	2.01<br>*2.36*	168.5	-	-
**b**	X = $COCH_3$, Y = Z = H	A = B = H	2.30	123.3	2.05<br>*2.31*	156.3	2.03<br>*2.28*	170.8	-	-
**c**	X = Y = $COOCH_3$, Z = H	A = B = H	2.58	108.1	2.00<br>*2.38*	158.0	1.97<br>*2.40*	168.1	-	-
**d**	X = Y = $COOCH_3$, Z = H	A = CH_3, B = H	2.64	108.8	2.07<br>*2.47*	145.7	2.07<br>*2.49*	165.0	-	-
**e**	X = Y = $COOCH_3$, Z = H	A = B = CH_3	3.12	96.7	2.19<br>*2.59*	140.6	2.55<br>*3.35*	169.3	-	-
**f**	X = $COOCH_3$, Y = Z = H	A = B = H	2.25	109.0	2.07<br>*2.29*	156.2	2.00<br>*2.35*	169.3	-	-
**g**	X = Y = $COOCH_3$, Z = Ph	A = B = H	2.38	104.3	2.01<br>*2.38*	156.5	1.97<br>*2.42*	167.3	-	-
**h**	X = Y = $COOCH_3$, Z = Ph	A = B = CH_3	2.50	101.6	2.27<br>*2.70*	136.6	2.71<br>*2.93*	172.9	-	-
**i**	X = Y = $COOCH_3$, Z = 4-MeOPh	A = B = H	2.37	103.4	2.02<br>*2.38*	155.6	1.98<br>*2.42*	167.2	-	-
**j**	X = Y = $COOCH_3$, Z = 4-MeOPh	A = B = CH_3	2.50	101.6	2.27<br>*2.69*	137.0	2.71<br>*2.93*	172.9	-	-
**k**	X = Y = $COOCH_3$, Z = 4-NO_2Ph	A = B = H	2.47	102.1	2.01<br>*2.39*	155.7	1.98<br>*2.42*	166.4	-	-
**l**	X = Y = $COOCH_3$, Z = 4-NO_2Ph	A = B = CH_3	2.55	101.8	2.28<br>*2.71*	136.8	2.70<br>*2.97*	166.7	-	-
**m**	X = Y = $CON(CH_3)_2$, Z = H	A = B = H	2.36	110.2	2.04<br>*2.31*	153.9	1.99<br>*2.38*	170.0	-	-
**n**	X = $COOCH_3$, Y = CN, Z = H	A = B = H	2.68	110.8	1.99<br>*2.40*	153.3	1.97<br>*2.41*	167.4	-	-

**o**	X = Y = CN, Z = H	A = B = H	3.05	106.5	1.99<br>*2.43*	157.2	-	-	-	-
**p**	X = CN, Y = Z = H	A = B = H	3.17	115.9	2.12<br>*2.28*	156.8	-	-	-	-
**q**	X = Y = NO_2, Z = H	A = B = H	No TS	No TS	1.89<br>*2.48*	155.2	-	-	1.91<br>*2.43*	170.3
**r**	X = NO_2, Y = Z = H	A = B = H	2.47	108.4	1.99<br>*2.36*	153.2	-	-	1.97<br>*2.33*	171.8
**s**	X = $COOCH_3$, Y = $PO(OMe_2)$, Z = H	A = B = H	3.15	98.7	2.01<br>*2.39*	157.6	1.95<br>*2.44*	170.8	1.97<br>*2.49*	167.7
**t**	X = (*E*)-CH=NPh, Y = CN, Z = H	A = B = H	2.90	106.9	1.98<br>*2.43*	153.2	-	-	2.07<br>*2.32*	171.3
**u**	X = $CSSCH_3$, Y= CN, Z = H	A = B = H	2.76	111.9	1.94<br>*2.49*	155.9	-	-	2.40<br>*2.40*	170.1

[a]Angle between C(phosphorane)-C(β-alkene)-C(α-alkene). [b]Angle between C(α to acceptors)-C(α to phosphorus)-P. [c]Angle between O-C(α to phosphorus)-P. [d]Angle between X (**q**,**r**,**s** : O; **t**: N; **u**: S) – C(α to phosphorus) – P.

Table S2: In normal font: Electronic energies, relative to sum of energies of **1** + **2**, calculated using DLPNO-CCSD(T)/def2-TZVP(CPCM(C),THF)//M06-2X/cc-pVDZ(pcm,THF). In *italics*: Gibbs free energies, relative to sum of energies of **1** + **2**, calculated using M06-2X/cc-pVDZ(pcm,THF).

System	Precursor **1**	Phosphorane **2**	TS **1**+**2**→**3**	**3**	TS **3**→**4**[a]	**4**[a]	TS **3**→**5**[a]	**5**[a]	TS **3**→(**6**-**9**)[a]	**6**-**9**[a]
a	X = Y = $COCH_3$, Z = H	A = B = H	1.7<br>*12.9*	-37.9<br>*-22.6*	-6.3<br>*-0.5*	-26.9<br>*-34.7*	-2.5<br>*5.4*	-34.0<br>*-40.6*	-	-
b	X = $COCH_3$, Y = Z = H	A = B = H	7.1<br>*14.8*	-20.2<br>*-6.3*	6.4<br>*10.3*	-26.0<br>*-36.6*	12.4<br>*22.5*	-26.5<br>*-33.1*	-	-
c	X = Y = $COOCH_3$, Z = H	A = B = H	0.5<br>*9.4*	-33.9<br>*-21.4*	-3.1<br>*0.5*	-28.1<br>*-37.5*	6.8<br>*12.4*	-18.8<br>*-26.7*	-	-
d	X = Y = $COOCH_3$, Z = H	A = CH_3, B = H	-1.9<br>*7.4*	-37.3<br>*-25.3*	-8.5<br>*-5.2*	-34.1<br>*-43.9*	2.1<br>*8.1*	-27.1<br>*-35.5*	-	-
e	X = Y = $COOCH_3$, Z = H	A = B = CH_3	-4.4	-37.0	-8.9	-36.5	-0.9	-33.7	-	-

			7.7	*-23.8*	*-5.4*	*-46.1*	*0.3*	*-41.8*	
f	X = $COOCH_3$, Y = Z = H	A = B = H	7.8<br>*15.3*	-13.5<br>*-1.7*	10.2<br>*13.8*	-25.3<br>*-35.1*	23.9<br>*29.9*	-7.5<br>*-14.9*	-	-
g	X = Y = $COOCH_3$, Z = Ph	A = B = H	1.9<br>*12.9*	-31.1<br>*-15.7*	-0.5<br>*5.6*	-26.9<br>*-35.9*	4.6<br>*14.6*	-16.9<br>*-24.3*	-	-
h	X = Y = $COOCH_3$, Z = Ph	A = B = CH_3	-2.2<br>*10.7*	-28.7<br>*-13.4*	-3.0<br>*1.8*	-32.8<br>*-42.7*	1.6<br>*5.2*	-32.3<br>*-38.7*	-	-
i	X = Y = $COOCH_3$, Z = 4-MeOPh	A = B = H	1.8<br>*12.8*	-30.4<br>*-14.8*	0.0<br>*6.2*	-26.3<br>*-35.2*	4.7<br>*15.6*	-16.0<br>*-23.2*	-	-
j	X = Y = $COOCH_3$, Z = 4-MeOPh	A = B = CH_3	-2.3<br>*11.2*	-27.3<br>*-12.0*	-3.5<br>*2.8*	-32.0<br>*-41.8*	2.2<br>*5.9*	-31.4<br>*-37.7*	-	-
k	X = Y = $COOCH_3$, Z = 4-NO_2Ph	A = B = H	-3.1<br>*10.2*	-32.8<br>*-17.6*	-1.9<br>*11.0*	-27.6<br>*-37.3*	1.1<br>*11.7*	-18.2<br>*-26.1*	-	-
l	X = Y = $COOCH_3$, Z = 4-NO_2Ph	A = B = CH_3	-4.6<br>*8.5*	-30.6<br>*-16.1*	-5.3<br>*0.3*	-33.8<br>*-44.1*	-1.1<br>*1.9*	-33.6<br>*-40.5*	-	-
m	X = Y = $CON(CH_3)_2$, Z = H	A = B = H	3.2<br>*13.7*	-21.6<br>*-8.9*	4.2<br>*9.5*	-27.5<br>*-36.9*	14.4<br>*22.2*	-16.7<br>*-23.6*	-	-
n	X = $COOCH_3$, Y = CN, Z = H	A = B = H	-1.5<br>*8.0*	-35.6<br>*-22.4*	-4.1<br>*-0.2*	-27.2<br>*-36.1*	6.7<br>*12.5*	-17.5<br>*-25.6*	-	-
o	X = Y = CN, Z = H	A = B = H	-4.6<br>*5.4*	-37.0<br>*-26.2*	-1.0<br>*-2.1*	-26.2<br>*-35.1*	-	-	-	-
p	X = CN, Y = Z = H	A = B = H	-1.7<br>*7.8*	-10.0<br>*2.9*	12.8<br>*15.4*	-25.2<br>*-34.7*	-	-	-	-
q	X = Y = NO_2, Z = H	A = B = H	No barrier	-53.4<br>*-42.0*	-9.7<br>*-9.3*	-30.3<br>*-40.6*	-	-	-10.6<br>*-6.4*	-33.9<br>*-41.6*
r	X = NO_2, Y = Z = H	A = B = H	2.1<br>*9.5*	-32.2<br>*-17.0*	1.8<br>*5.8*	-27.1<br>*-37.0*	-	-	3.4<br>*12.3*	-28.7<br>*-34.0*
s	X = $COOCH_3$, Y = PO(OMe_2), Z = H	A = B = H	-1.9<br>*9.1*	-32.9<br>*-20.6*	-3.6<br>*2.0*	-28.8<br>*-37.8*	8.5<br>*15.4*	-17.8<br>*-24.3*	14.6<br>*19.3*	-5.4<br>*-17.7*
t	X = (*E*)-CH=NPh, Y = CN, Z = H	A = B = H	-0.4<br>*9.6*	-35.7<br>*-25.7*	-2.5<br>*0.3*	-24.1<br>*-32.9*	-	-	0.7<br>*5.6*	-41.6<br>*-49.8*
u	X = $CSSCH_3$, Y = CN, Z = H	A = B = H	2.2	-47.6	-9.3	-27.1	-	-	-11.9	-44.4

			12.5	*-35.8*	*-6.7*	*-35.4*			*-7.6*	*-48.0*

[a] plus triphenylphosphine

Table S3: CDFT parameters calculated for alkenes **1** and Wittig ylides, based on M06-2X/cc-pVDZ(THF)

Alkene	Electronic chemical potential μ [eV]	Hardness η [eV]	Electrophilicity parameter ω [eV]	Nucleophilicity parameter *N* [eV]
1a	-4.91	7.66	1.57	1.59
1b	-4.65	8.37	1.29	1.49
1c	-5.02	8.56	1.47	1.04
1f	-4.80	9.17	1.26	0.94
1g	-4.63	6.90	1.55	2.25
1i	-4.25	6.39	1.41	2.88
1k	-5.29	6.64	2.11	1.72
1m	-4.17	8.10	1.07	2.11
1n	-5.41	8.48	1.73	0.68
1o	-5.66	8.33	1.92	0.51
1p	-4.88	9.04	1.32	0.93
1q	-6.21	8.36	2.31	-0.06
1r	-5.70	8.85	1.84	0.20
1s	-5.10	8.78	1.48	0.84
1t	-4.59	6.57	1.62	2.46
1u	-5.01	5.90	2.13	2.36
2A	-3.19	6.13	0.83	4.07
2B	-2.92	5.64	0.76	4.59
2C	-2.81	5.30	0.74	4.87

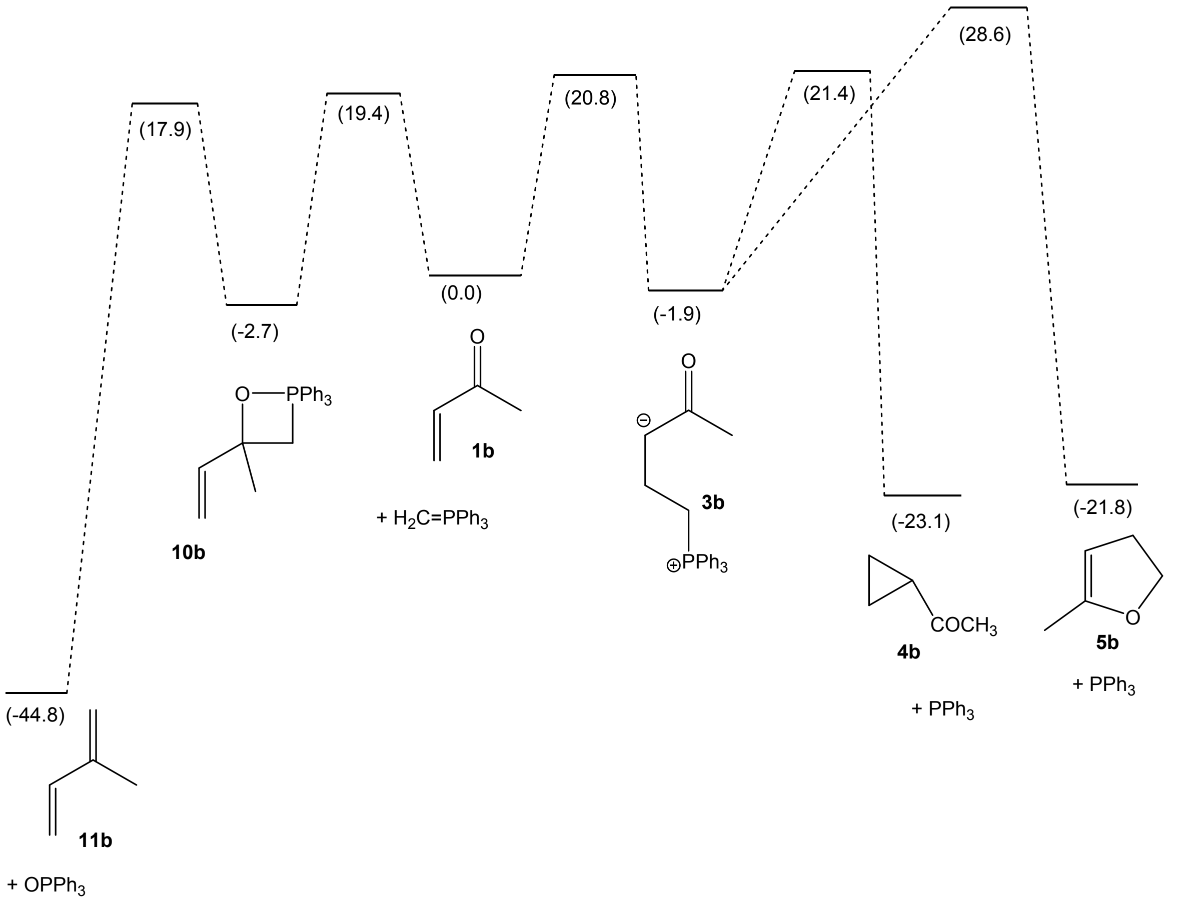

Figure S1: Schematic representation of the PES for the reaction of **1b** with triphenylphosphonium methylide. Gibbs free energies based on DLPNO-CCSD(T)/def2-TZVP(CPCM(C),THF)//M06-2X/cc-pVDZ(THF) electronic energies, with enthalpy and entropy correction from M06-2X/cc-pVDZ(THF), in kcal mol^{-1}, relative to **1b** + CH_2=PPh_3.

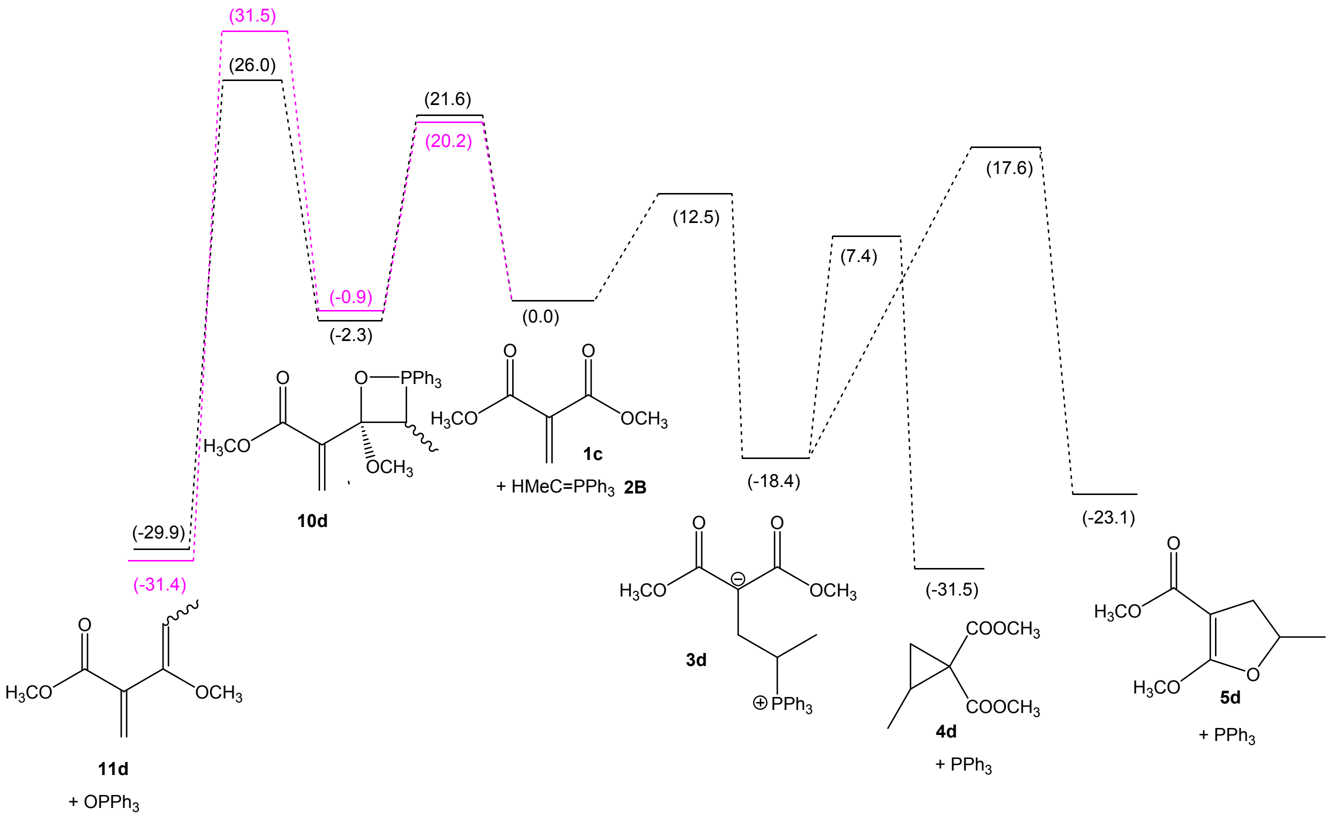

Figure S2: Schematic representation of the PES for the reaction of **1c** with triphenylphosphonium ethylide. Gibbs free energies based on DLPNO-CCSD(T)/def2-TZVP(CPCM(C),THF)//M06-2X/cc-pVDZ(THF) electronic energies, with enthalpy and entropy correction from M06-2X/cc-pVDZ(THF) in kcal mol^{-1}, relative to **1c** + $CHMe=PPh_3$. On the lefthand side, values entered in black refer to the pathway resulting in *E*-**11d**, values entered in magenta refer to the pathway yielding *Z*-**11d**.

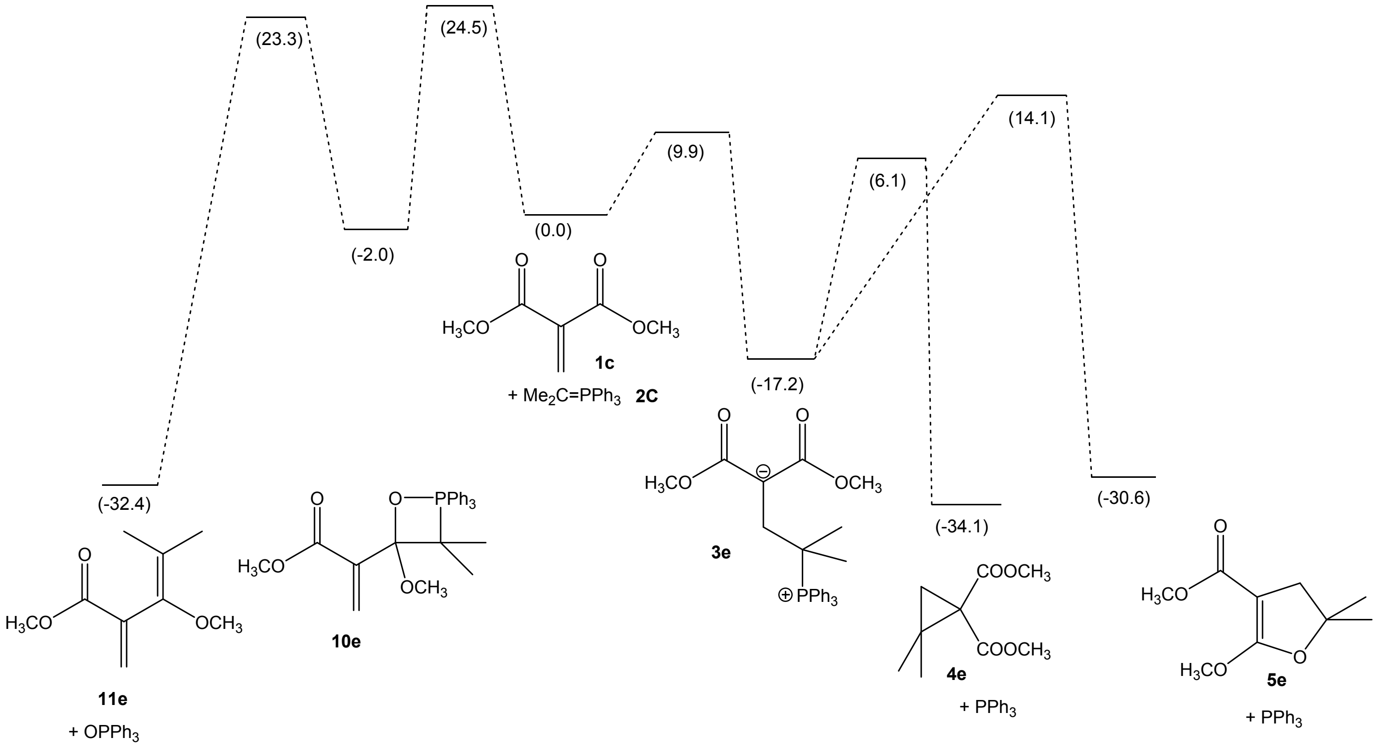

Figure S3: Schematic representation of the PES for the reaction of **1c** with triphenylphosphonium isopropylide. Gibbs free energies based on DLPNO-CCSD(T)/def2-TZVP(CPCM(C),THF)//M06-2X/cc-pVDZ(THF) electronic energies, with enthalpy and entropy correction from M06-2X/cc-pVDZ(THF) in kcal mol^{-1}, relative to **1c** + CMe_2=PPh_3.

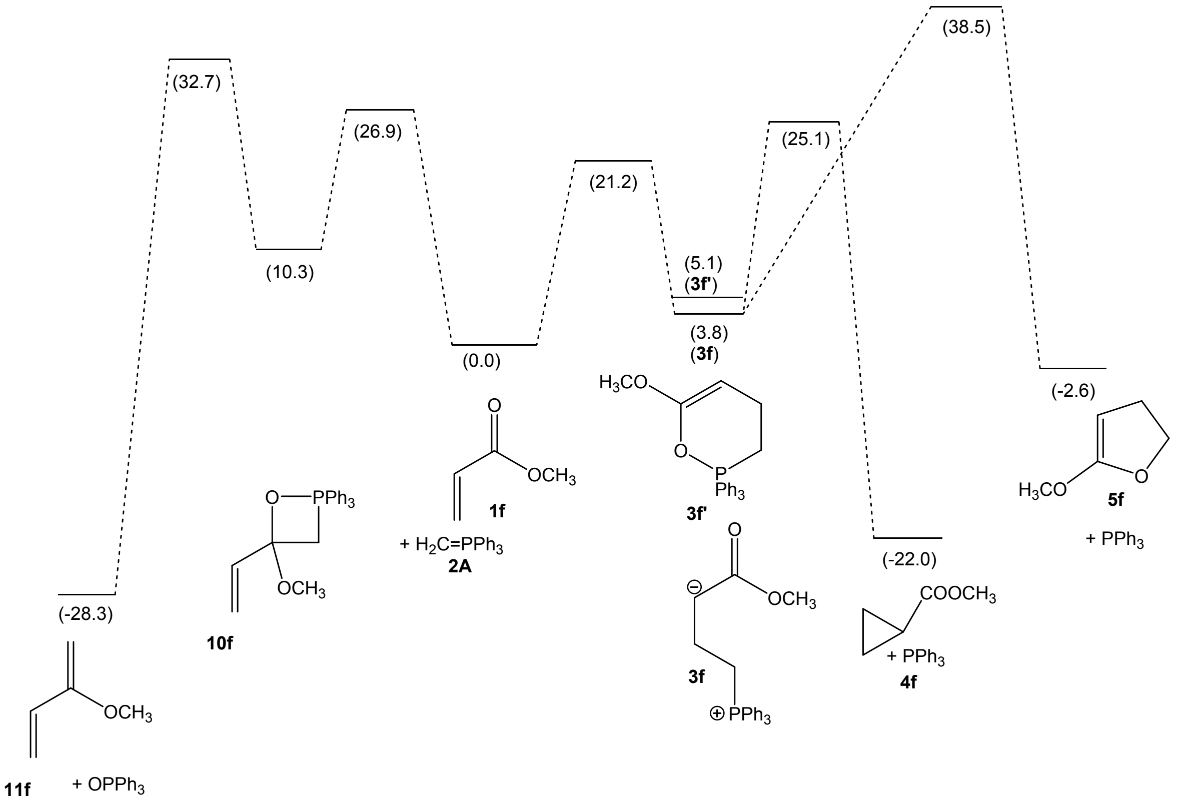

Figure S4: Schematic representation of the PES for the reaction of **1f** with triphenylphosphonium methylide. Gibbs free energies based on DLPNO-CCSD(T)/def2-TZVP(CPCM(C),THF)//M06-2X/cc-pVDZ(THF) electronic energies, with enthalpy and entropy correction from M06-2X/cc-pVDZ(THF) in kcal mol^{-1}, relative to **1f** + CH_2=PPh_3.

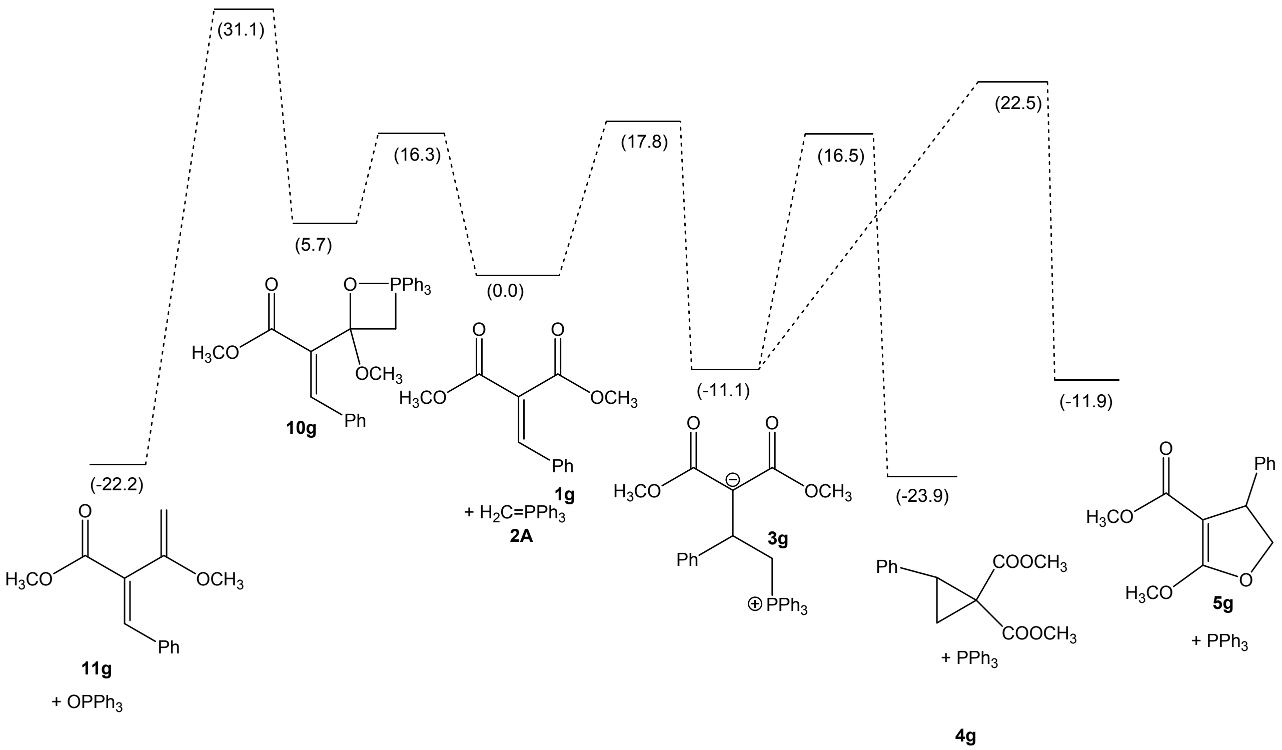

Figure S5: Schematic representation of the PES for the reaction of **1g** with triphenylphosphonium methylide. Gibbs free energies based on DLPNO-CCSD(T)/def2-TZVP(CPCM(C),THF)//M06-2X/cc-pVDZ(THF) electronic energies, with enthalpy and entropy correction from M06-2X/cc-pVDZ(THF) in kcal mol^{-1}, relative to **1g** + CH_2=PPh_3.

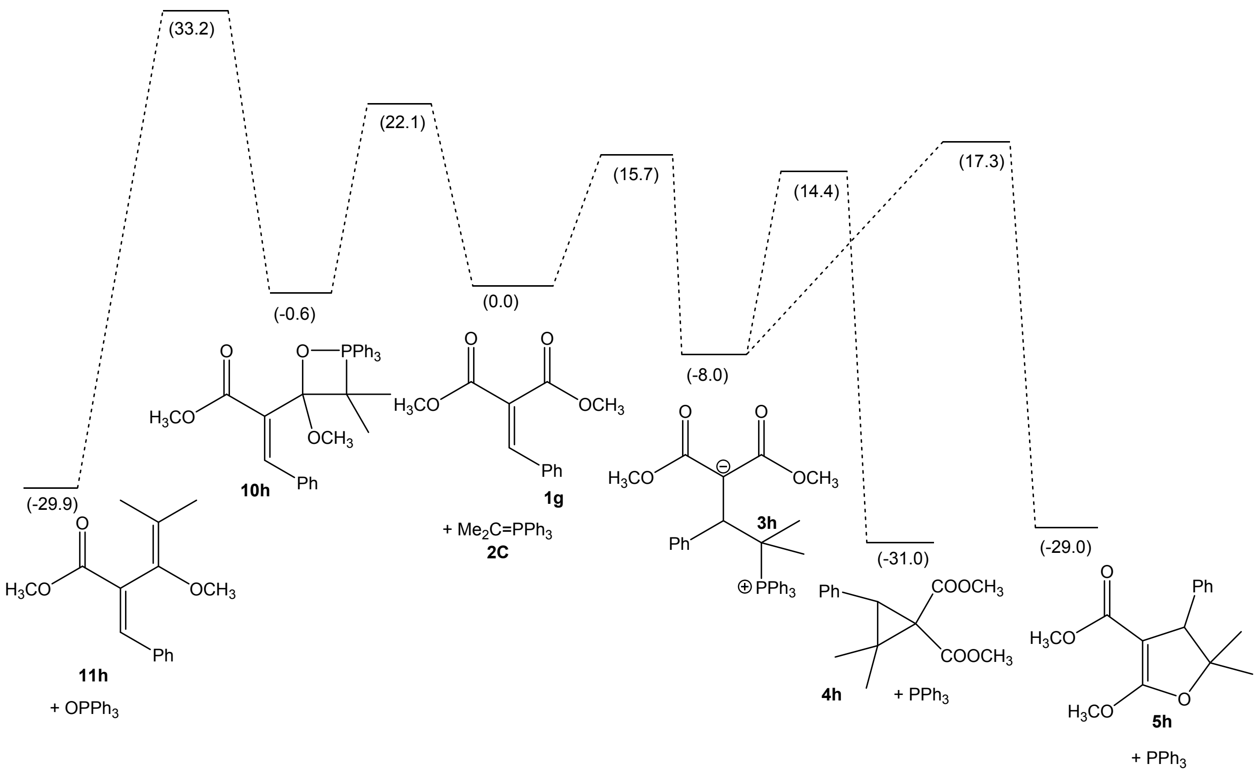

Figure S6: Schematic representation of the PES for the reaction of **1g** with triphenylphosphonium isopropylide. Gibbs free energies based on DLPNO-CCSD(T)/def2-TZVP(CPCM(C),THF)//M06-2X/cc-pVDZ(THF) electronic energies, with enthalpy and entropy correction from M06-2X/cc-pVDZ(THF) in kcal mol^{-1}, relative to **1g** + CMe_2=PPh_3.

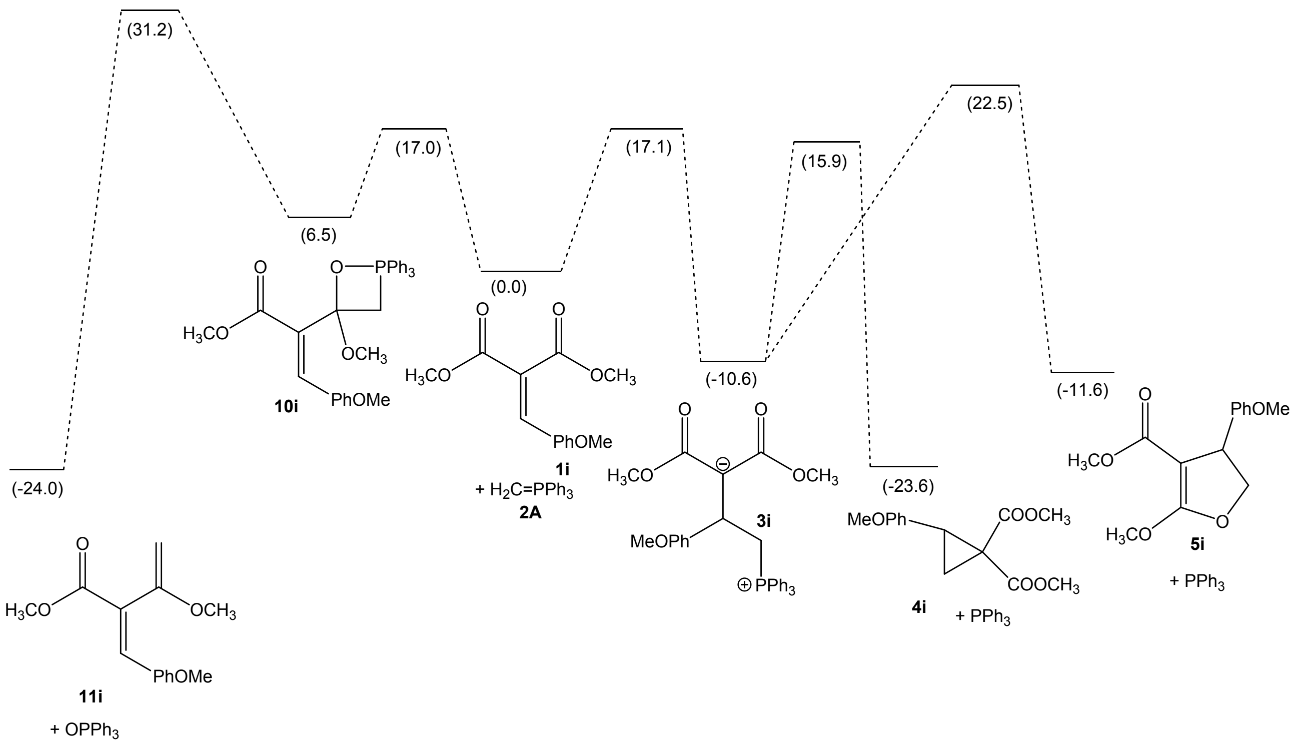

Figure S7: Schematic representation of the PES for the reaction of **1i** with triphenylphosphonium methylide. Gibbs free energies based on DLPNO-CCSD(T)/def2-TZVP(CPCM(C),THF)//M06-2X/cc-pVDZ(THF) electronic energies, with enthalpy and entropy correction from M06-2X/cc-pVDZ(THF) in kcal mol^{-1}, relative to **1i** + CH_2=PPh_3. PhOMe = 4-methoxyphenyl.

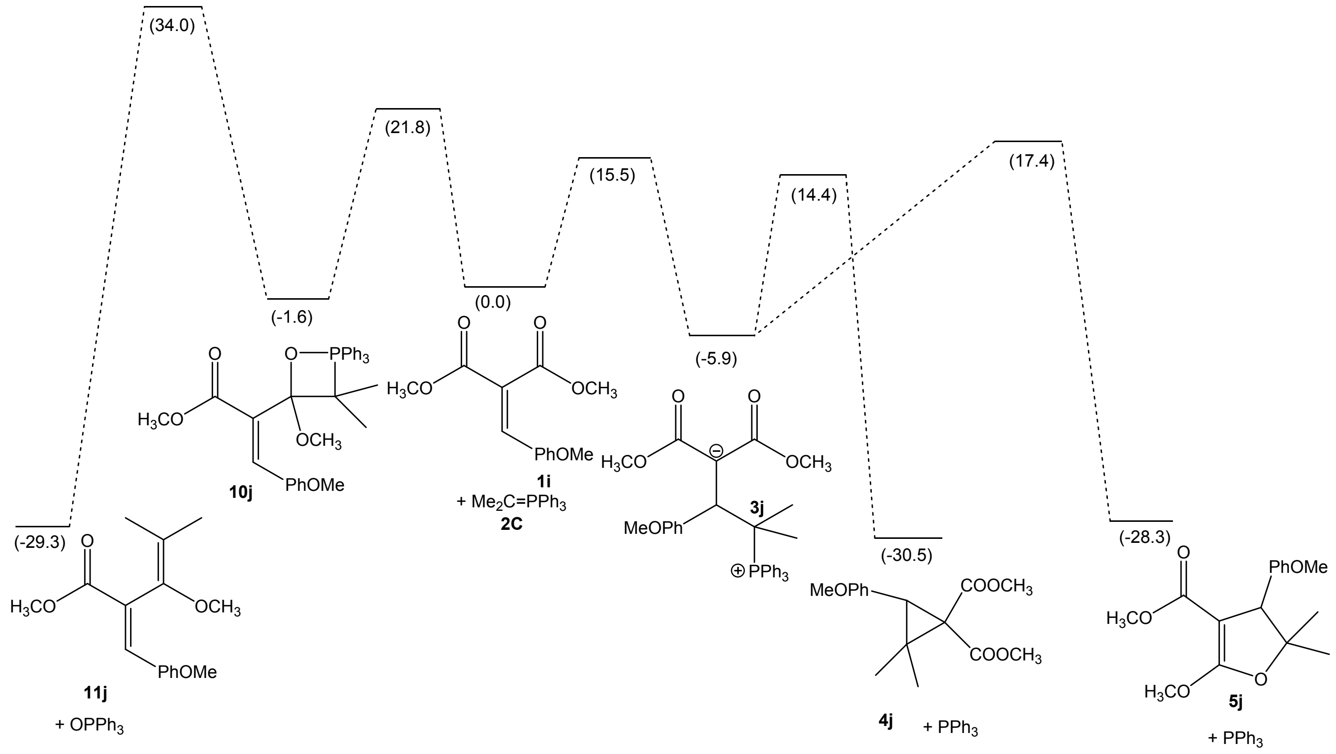

Figure S8: Schematic representation of the PES for the reaction of **1i** with triphenylphosphonium isopropylide. Gibbs free energies based on DLPNO-CCSD(T)/def2-TZVP(CPCM(C),THF)//M06-2X/cc-pVDZ(THF) electronic energies, with enthalpy and entropy correction from M06-2X/cc-pVDZ(THF) in kcal mol^{-1}, relative to **1i** + CMe_2=PPh_3. PhOMe = 4-methoxyphenyl.

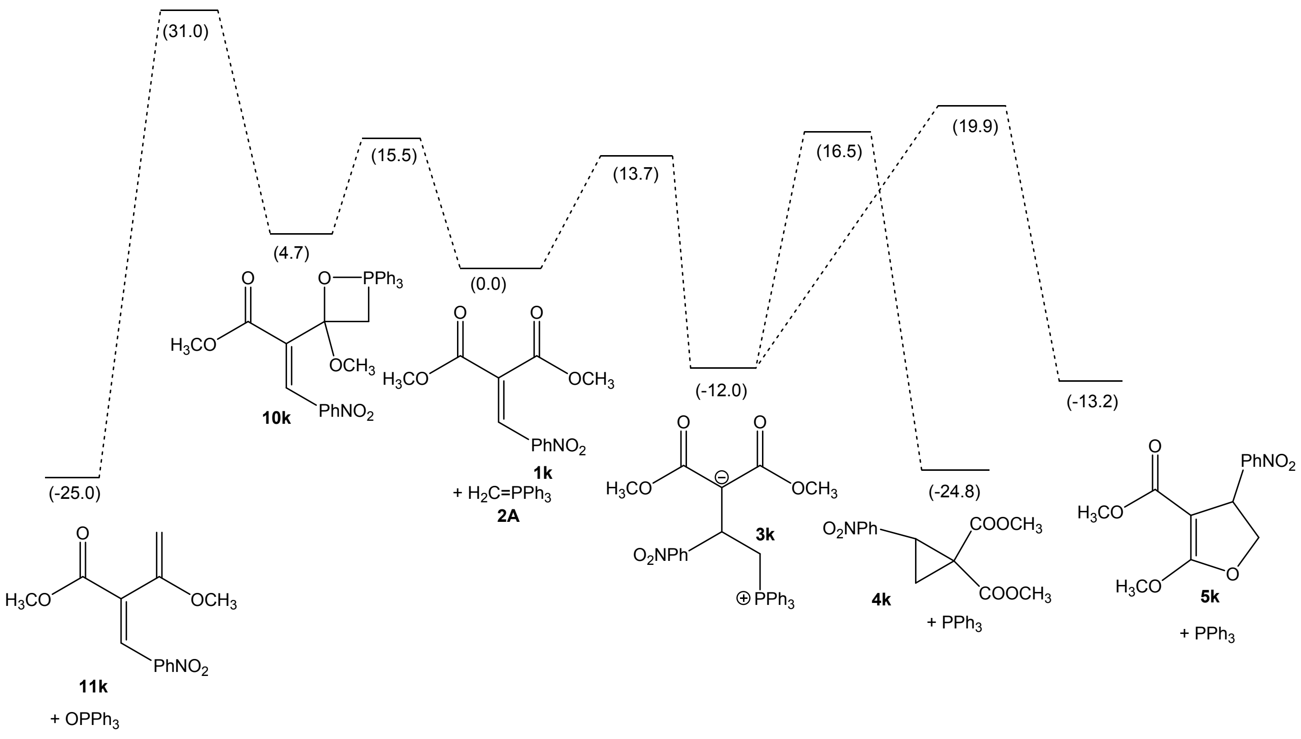

Figure S9: Schematic representation of the PES for the reaction of **1k** with triphenylphosphonium methylide. Gibbs free energies based on DLPNO-CCSD(T)/def2-TZVP(CPCM(C),THF)//M06-2X/cc-pVDZ(THF) electronic energies, with enthalpy and entropy correction from M06-2X/cc-pVDZ(THF) in kcal mol^{-1}, relative to **1k** + CH_2=PPh_3. $PhNO_2$ = 4-nitrophenyl.

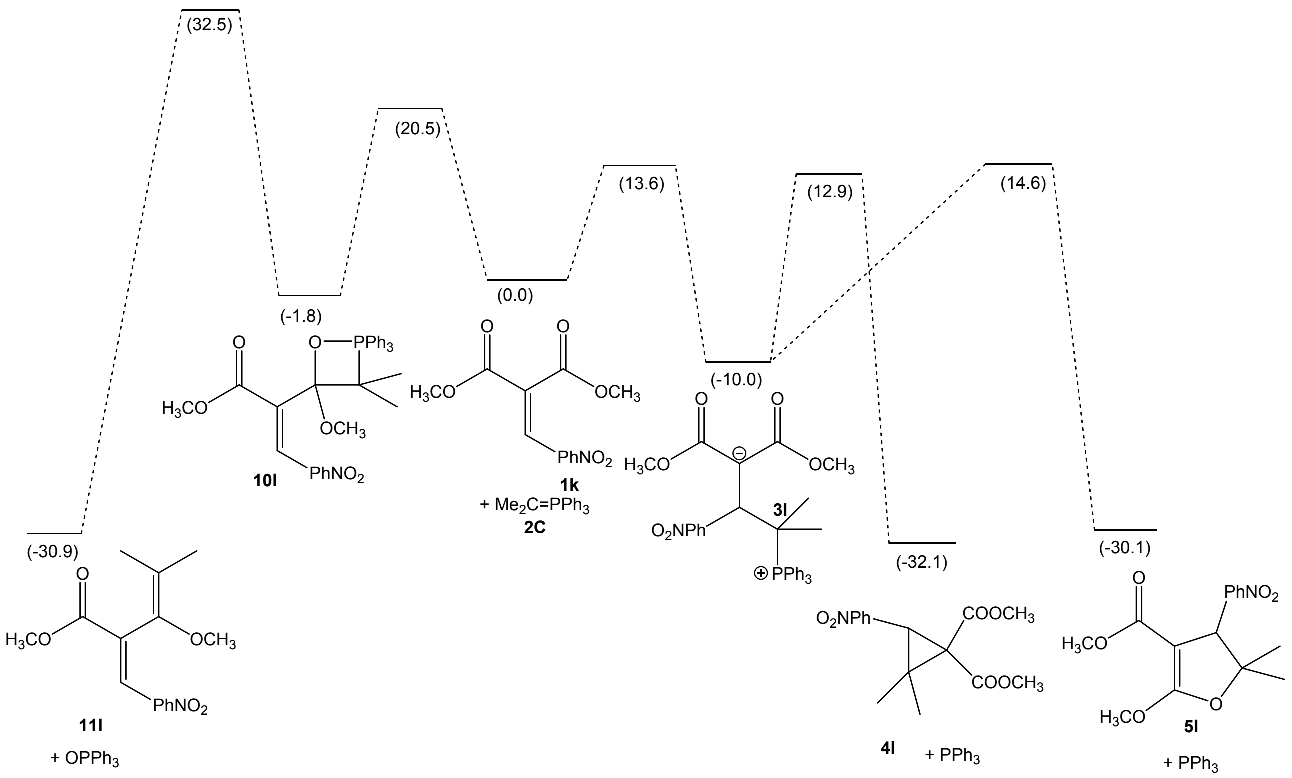

Figure S10: Schematic representation of the PES for the reaction of **1l** with triphenylphosphonium isopropylide. Gibbs free energies based on DLPNO-CCSD(T)/def2-TZVP(CPCM(C),THF)//M06-2X/cc-pVDZ(THF) electronic energies, with enthalpy and entropy correction from M06-2X/cc-pVDZ(THF) in kcal mol^{-1}, relative to **1k** + CMe_2=PPh_3. $PhNO_2$ = 4-nitrophenyl.

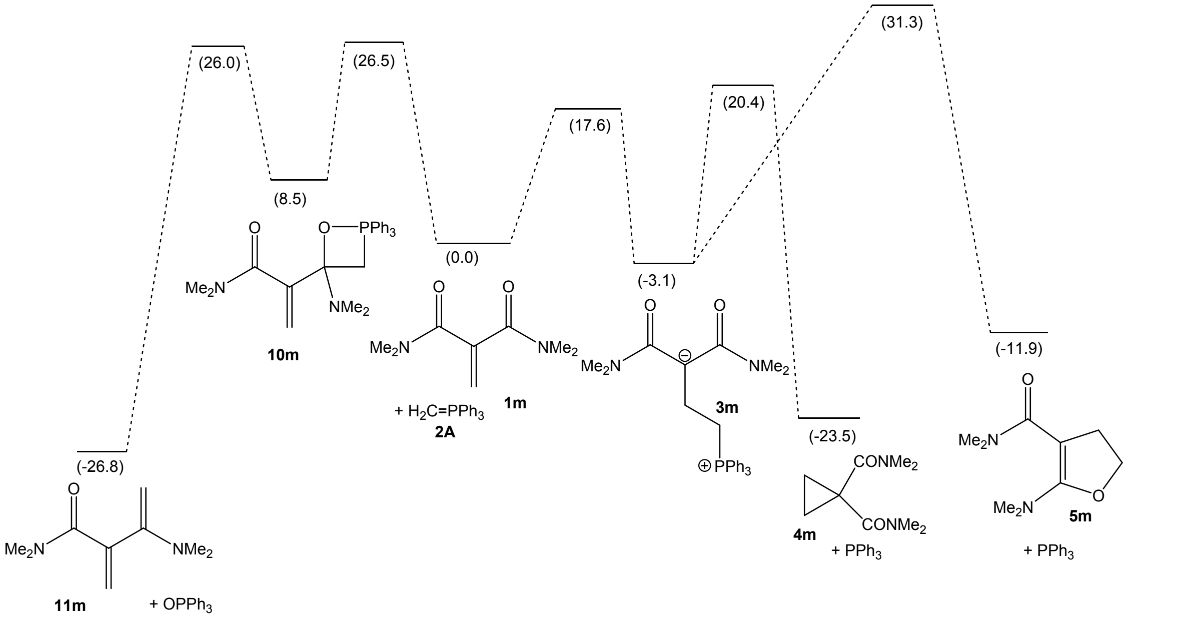

Figure S11: Schematic representation of the PES for the reaction of **1m** with triphenylphosphonium methylide. Gibbs free energies based on DLPNO-CCSD(T)/def2-TZVP(CPCM(C),THF)//M06-2X/cc-pVDZ(THF) electronic energies, with enthalpy and entropy correction from M06-2X/cc-pVDZ(THF) in kcal mol^{-1}, relative to **1m** + CH_2=PPh_3.

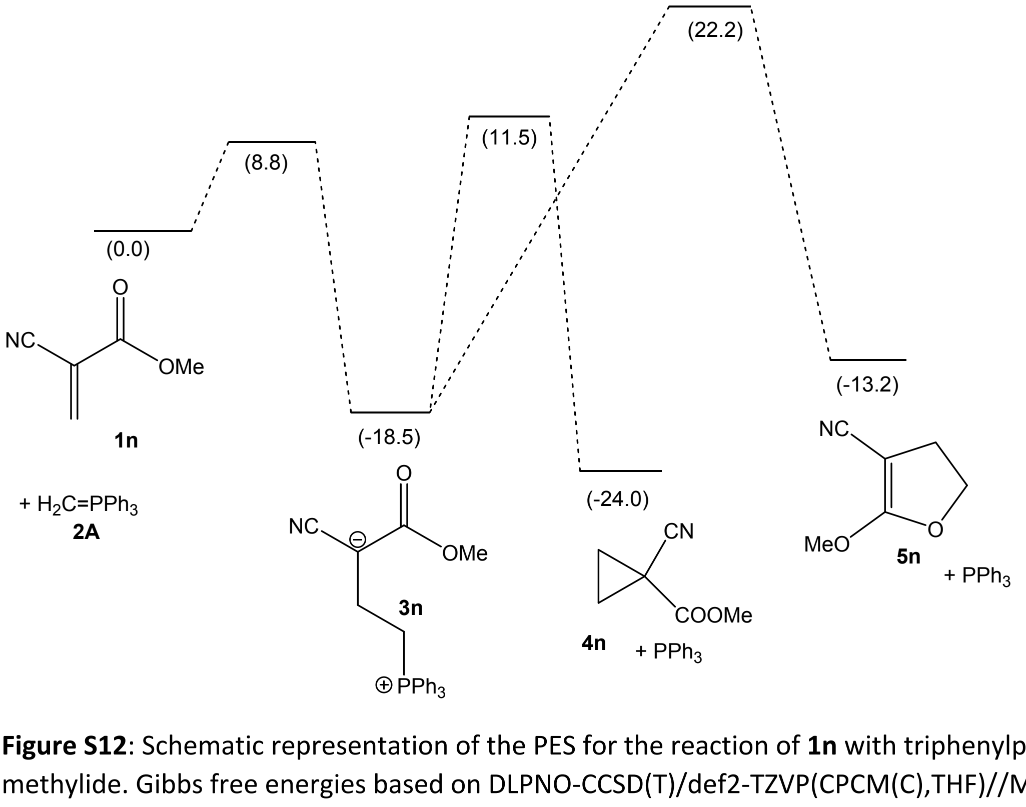

Figure S12: Schematic representation of the PES for the reaction of **1n** with triphenylphosphonium methylide. Gibbs free energies based on DLPNO-CCSD(T)/def2-TZVP(CPCM(C),THF)//M06-2X/cc-pVDZ(THF) electronic energies, with enthalpy and entropy correction from M06-2X/cc-pVDZ(THF) in kcal mol^{-1}, relative to **1n** + CH_2=PPh_3. Only the conjugate addition pathway was considered in this system.

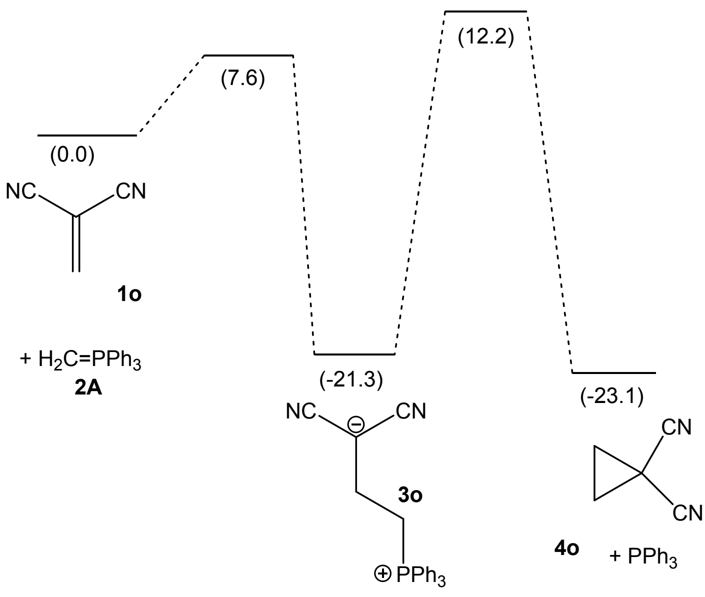

Figure S13: Schematic representation of the PES for the reaction of **1o** with triphenylphosphonium methylide. Gibbs free energies based on DLPNO-CCSD(T)/def2-TZVP(CPCM(C),THF)//M06-2X/cc-pVDZ(THF) electronic energies, with enthalpy and entropy correction from M06-2X/cc-pVDZ(THF) in kcal mol^{-1}, relative to **1o** + CH_2=PPh_3.

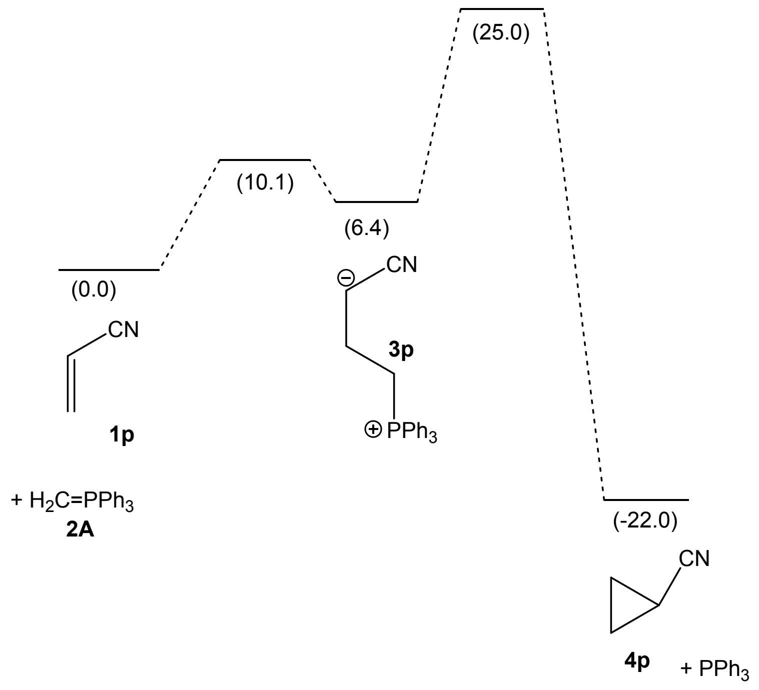

Figure S14: Schematic representation of the PES for the reaction of **1p** with triphenylphosphonium methylide. Gibbs free energies based on DLPNO-CCSD(T)/def2-TZVP(CPCM(C),THF)//M06-2X/cc-pVDZ(THF) electronic energies, with enthalpy and entropy correction from M06-2X/cc-pVDZ(THF) in kcal mol^{-1}, relative to **1p** + CH_2=PPh_3.

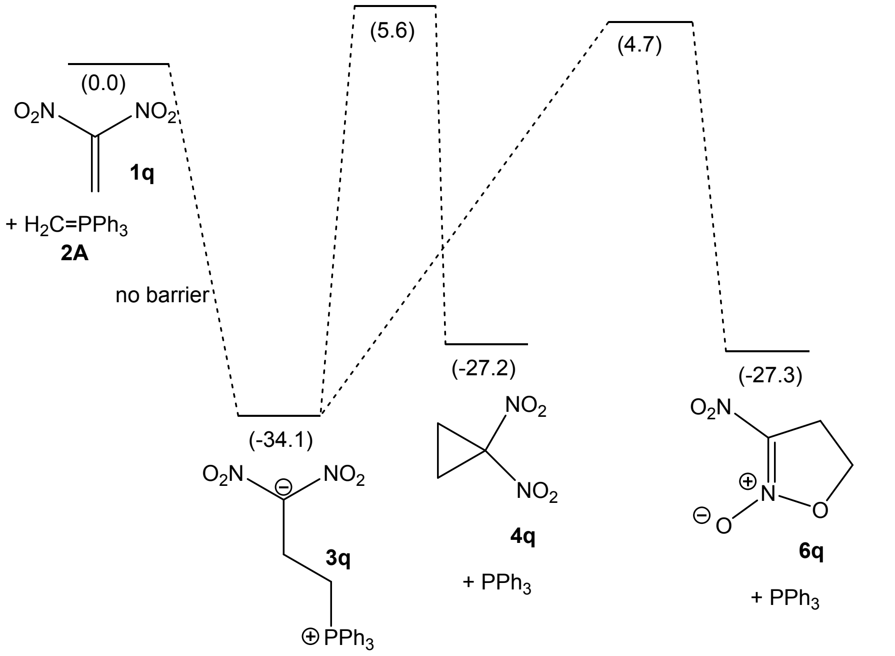

Figure S15: Schematic representation of the PES for the reaction of **1q** with triphenylphosphonium methylide. Gibbs free energies based on DLPNO-CCSD(T)/def2-TZVP(CPCM(C),THF)//M06-2X/cc-pVDZ(THF) electronic energies, with enthalpy and entropy correction from M06-2X/cc-pVDZ(THF) in kcal mol^{-1}, relative to **1q** + CH_2=PPh_3.

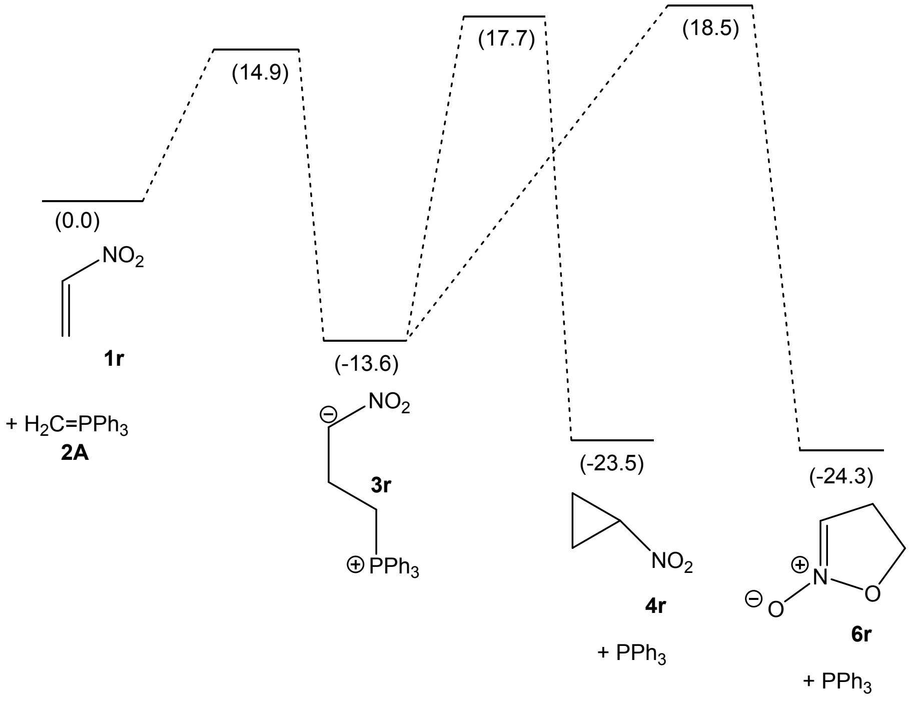

Figure S16: Schematic representation of the PES for the reaction of **1r** with triphenylphosphonium methylide. Gibbs free energies based on DLPNO-CCSD(T)/def2-TZVP(CPCM(C),THF)//M06-2X/cc-pVDZ(THF) electronic energies, with enthalpy and entropy correction from M06-2X/cc-pVDZ(THF) in kcal mol^{-1}, relative to **1r** + CH_2=PPh_3.

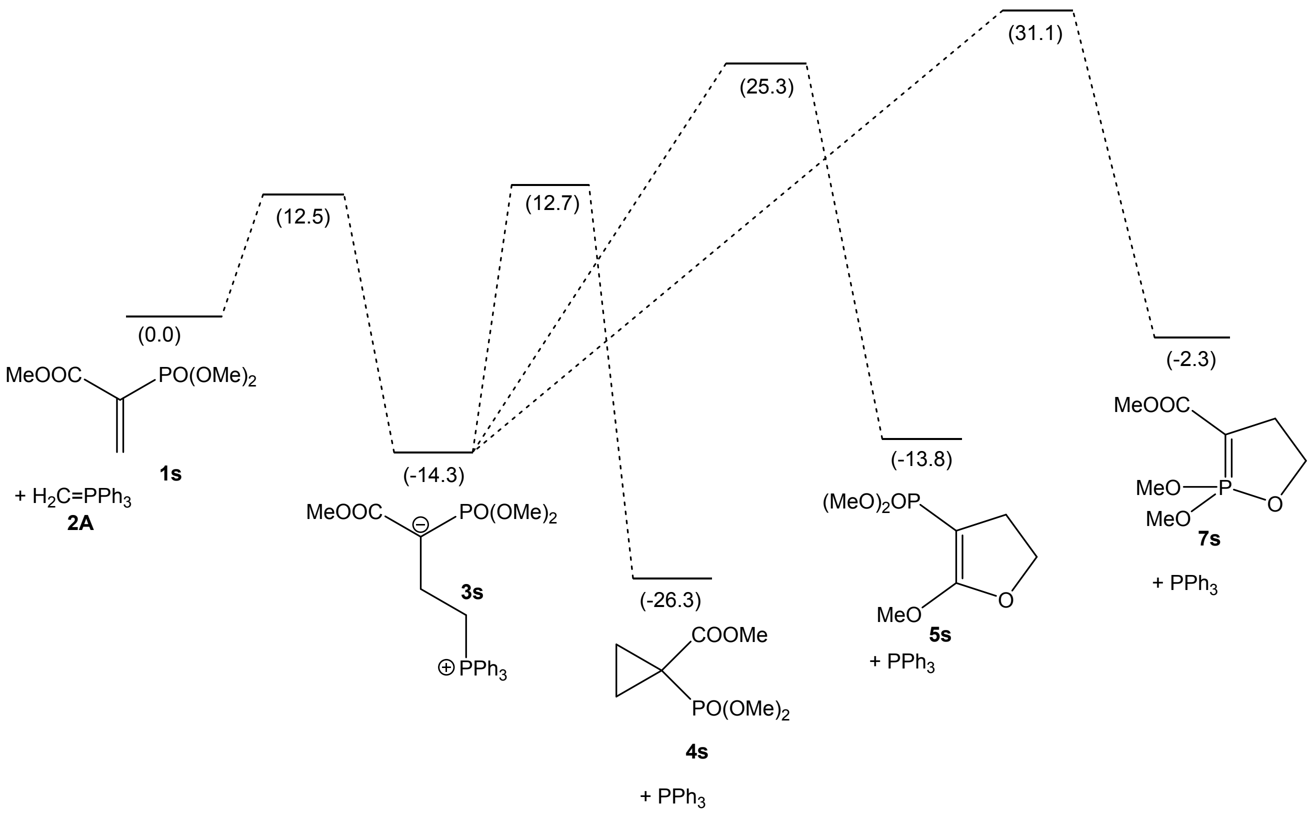

Figure S17: Schematic representation of the PES for the reaction of **1s** with triphenylphosphonium methylide. Gibbs free energies based on DLPNO-CCSD(T)/def2-TZVP(CPCM(C),THF)//M06-2X/cc-pVDZ(THF) electronic energies, with enthalpy and entropy correction from M06-2X/cc-pVDZ(THF) in kcal mol^{-1}, relative to **1s** + CH_2=PPh_3. Only the conjugate addition pathway was considered in this system.

Methylenephosphorane 2A
M06-2X/cc-pVDZ(pcm,THF): -1075.2959784
DLPNO-CCSD(T)/def2-TZVP(CPCM(C),THF)// M06-2X/cc-pVDZ(pcm,THF): -1073.701773

Ethylidenephosphorane 2B
M06-2X/cc-pVDZ(pcm,THF): -1114.585116
DLPNO-CCSD(T)/def2-TZVP(CPCM(C),THF)// M06-2X/cc-pVDZ(pcm,THF): -1112.930383

Isopropylidenephosphorane 2C
M06-2X/cc-pVDZ(pcm,THF): -1153.8779237
DLPNO-CCSD(T)/def2-TZVP(CPCM(C),THF)// M06-2X/cc-pVDZ(pcm,THF): -1152.162295

Triphenylphosphine
M06-2X/cc-pVDZ(pcm,THF): -1036.0614129
DLPNO-CCSD(T)/def2-TZVP(CPCM(C),THF)// M06-2X/cc-pVDZ(pcm,THF): -1034.514264

Triphenylphosphine oxide

M06-2X/cc-pVDZ(pcm,THF): -1111.295345
DLPNO-CCSD(T)/def2-TZVP(CPCM(C),THF)// M06-2X/cc-pVDZ(pcm,THF): -1109.696288

Triphenylphosphine sulfide
M06-2X/cc-pVDZ(pcm,THF): -1434.2699655
DLPNO-CCSD(T)/def2-TZVP(CPCM(C),THF)// M06-2X/cc-pVDZ(pcm,THF): -1432.288326

Triphenylphosphine *N*-phenylimide
M06-2X/cc-pVDZ(pcm,THF): -1322.3679102
DLPNO-CCSD(T)/def2-TZVP(CPCM(C),THF)// M06-2X/cc-pVDZ(pcm,THF): -1320.394136

Methyleneacetylacetone 1a
M06-2X/cc-pVDZ(pcm,THF): -383.7496719
DLPNO-CCSD(T)/def2-TZVP(CPCM(C),THF)// M06-2X/cc-pVDZ(pcm,THF): -383.238110

TS for reaction of 1a + 2A -> 3a
M06-2X/cc-pVDZ(pcm,THF): -1459.0481592
DLPNO-CCSD(T)/def2-TZVP(CPCM(C),THF)// M06-2X/cc-pVDZ(pcm,THF): -1456.937179

Zwitterion 3a
M06-2X/cc-pVDZ(pcm,THF): -1459.1120691
DLPNO-CCSD(T)/def2-TZVP(CPCM(C),THF)// M06-2X/cc-pVDZ(pcm,THF): -1457.000340

TS 3a -> 4a + PPh_3
M06-2X/cc-pVDZ(pcm,THF): -1459.0733799
DLPNO-CCSD(T)/def2-TZVP(CPCM(C),THF)// M06-2X/cc-pVDZ(pcm,THF): -1456.949925

1,1-Diacetylcyclopropane 4a
M06-2X/cc-pVDZ(pcm,THF): -423.0455504
DLPNO-CCSD(T)/def2-TZVP(CPCM(C),THF)// M06-2X/cc-pVDZ(pcm,THF): -422.468421

TS 3a -> 5a + PPh_3
M06-2X/cc-pVDZ(pcm,THF): -1459.0620715
DLPNO-CCSD(T)/def2-TZVP(CPCM(C),THF)// M06-2X/cc-pVDZ(pcm,THF): -1456.943943

4*H*,5*H*-2-methyl-3-acetylfuran 5a
M06-2X/cc-pVDZ(pcm,THF): -423.0563808
DLPNO-CCSD(T)/def2-TZVP(CPCM(C),THF)// M06-2X/cc-pVDZ(pcm,THF): -422.479791

TS 1a + 2A -> 10a
M06-2X/cc-pVDZ(pcm,THF): -1459.0495577
DLPNO-CCSD(T)/def2-TZVP(CPCM(C),THF)// M06-2X/cc-pVDZ(pcm,THF): -1456.932289

Oxaphosphetane 10a
M06-2X/cc-pVDZ(pcm,THF): -1459.0878988
DLPNO-CCSD(T)/def2-TZVP(CPCM(C),THF)// M06-2X/cc-pVDZ(pcm,THF): -1456.9763447

TS 10a -> 11a + $O{=}PPh_3$
M06-2X/cc-pVDZ(pcm,THF): -1459.041922
DLPNO-CCSD(T)/def2-TZVP(CPCM(C),THF)// M06-2X/cc-pVDZ(pcm,THF): -1456.9373358

2-Acetyl-3-methyl-1,3-butadiene 11a
M06-2X/cc-pVDZ(pcm,THF): -347.8162062
DLPNO-CCSD(T)/def2-TZVP(CPCM(C),THF)// M06-2X/cc-pVDZ(pcm,THF): -347.320632

Methylvinylketone 1b
M06-2X/cc-pVDZ(pcm,THF): -231.1495154
DLPNO-CCSD(T)/def2-TZVP(CPCM(C),THF)// M06-2X/cc-pVDZ(pcm,THF): -230.838206

TS 1b + 2A -> 3b
M06-2X/cc-pVDZ(pcm,THF): -1306.4437256
DLPNO-CCSD(T)/def2-TZVP(CPCM(C),THF)// M06-2X/cc-pVDZ(pcm,THF): -1304.528712

Zwitterion 3b
M06-2X/cc-pVDZ(pcm,THF): -1306.4847012
DLPNO-CCSD(T)/def2-TZVP(CPCM(C),THF)// M06-2X/cc-pVDZ(pcm,THF): -1304.572179

TS 3b -> 4b + PPh_3
M06-2X/cc-pVDZ(pcm,THF): -1306.4530957
DLPNO-CCSD(T)/def2-TZVP(CPCM(C),THF)// M06-2X/cc-pVDZ(pcm,THF): -1304.529813

Cyclopropylmethylketone 4b
M06-2X/cc-pVDZ(pcm,THF): -270.4471763
DLPNO-CCSD(T)/def2-TZVP(CPCM(C),THF)// M06-2X/cc-pVDZ(pcm,THF): -270.067217

TS 3b -> 5b + PPh_3
M06-2X/cc-pVDZ(pcm,THF): -1306.4375823
DLPNO-CCSD(T)/def2-TZVP(CPCM(C),THF)// M06-2X/cc-pVDZ(pcm,THF): -1304.520210

4*H*,5*H*-2-methylfuran 5b
M06-2X/cc-pVDZ(pcm,THF): -270.4443226
DLPNO-CCSD(T)/def2-TZVP(CPCM(C),THF)// M06-2X/cc-pVDZ(pcm,THF): -270.067864

TS 1b + 2A -> 10b
M06-2X/cc-pVDZ(pcm,THF): -1306.4461891
DLPNO-CCSD(T)/def2-TZVP(CPCM(C),THF)// M06-2X/cc-pVDZ(pcm,THF): -1304.534675

Oxaphosphetane 10b
M06-2X/cc-pVDZ(pcm,THF): -1306.4802845
DLPNO-CCSD(T)/def2-TZVP(CPCM(C),THF)// M06-2X/cc-pVDZ(pcm,THF): -1304.573975

TS 10b -> 11b + O=PPh_3
M06-2X/cc-pVDZ(pcm,THF): -1306.438628
DLPNO-CCSD(T)/def2-TZVP(CPCM(C),THF)// M06-2X/cc-pVDZ(pcm,THF): -1304.538514

Isoprene 11b
M06-2X/cc-pVDZ(pcm,THF): -195.2210194
DLPNO-CCSD(T)/def2-TZVP(CPCM(C),THF)// M06-2X/cc-pVDZ(pcm,THF): -194.922161

Dimethylmethylenemalonate 1c
M06-2X/cc-pVDZ(pcm,THF): -534.1791027
DLPNO-CCSD(T)/def2-TZVP(CPCM(C),THF)// M06-2X/cc-pVDZ(pcm,THF): -533.517399

TS 1c + 2A -> 3c
M06-2X/cc-pVDZ(pcm,THF): -1609.4824236
DLPNO-CCSD(T)/def2-TZVP(CPCM(C),THF)// M06-2X/cc-pVDZ(pcm,THF): -1607.218363

Zwitterion 3c
M06-2X/cc-pVDZ(pcm,THF): -1609.5391852
DLPNO-CCSD(T)/def2-TZVP(CPCM(C),THF)// M06-2X/cc-pVDZ(pcm,THF): -1607.273131

TS 3c -> 4c + PPh_3
M06-2X/cc-pVDZ(pcm,THF): -1609.4668063
DLPNO-CCSD(T)/def2-TZVP(CPCM(C),THF)// M06-2X/cc-pVDZ(pcm,THF): -1607.224059

Cyclopropane 4c
M06-2X/cc-pVDZ(pcm,THF): -573.4785647
DLPNO-CCSD(T)/def2-TZVP(CPCM(C),THF)// M06-2X/cc-pVDZ(pcm,THF): -572.749693

TS 3c -> 5c + PPh_3
M06-2X/cc-pVDZ(pcm,THF): -1609.4993584
DLPNO-CCSD(T)/def2-TZVP(CPCM(C),THF)// M06-2X/cc-pVDZ(pcm,THF): -1607.208280

Dihydrofuran 5c
M06-2X/cc-pVDZ(pcm,THF): -573.4633317
DLPNO-CCSD(T)/def2-TZVP(CPCM(C),THF)// M06-2X/cc-pVDZ(pcm,THF): -572.734912

TS 1c + 2A -> 10c
M06-2X/cc-pVDZ(pcm,THF): -1609.4743242
DLPNO-CCSD(T)/def2-TZVP(CPCM(C),THF)// M06-2X/cc-pVDZ(pcm,THF): -1607.206489

Oxaphosphetane 10c
M06-2X/cc-pVDZ(pcm,THF): -1609.5044487
DLPNO-CCSD(T)/def2-TZVP(CPCM(C),THF)// M06-2X/cc-pVDZ(pcm,THF): -1607.237693

TS 10c -> 11c + O=PPh_3
M06-2X/cc-pVDZ(pcm,THF): -1609.454352
DLPNO-CCSD(T)/def2-TZVP(CPCM(C),THF)// M06-2X/cc-pVDZ(pcm,THF): -1607.190311

2-Methoxycarbonyl-3-methoxy-1,2-butadiene 11c
M06-2X/cc-pVDZ(pcm,THF): -498.2181829
DLPNO-CCSD(T)/def2-TZVP(CPCM(C),THF)// M06-2X/cc-pVDZ(pcm,THF): -497.570065

TS 1c + 2B -> 3d
M06-2X/cc-pVDZ(pcm,THF): -1648.7754058
DLPNO-CCSD(T)/def2-TZVP(CPCM(C),THF)// M06-2X/cc-pVDZ(pcm,THF): -1646.450883

Zwitterion 3d
M06-2X/cc-pVDZ(pcm,THF): -1648.8347202
DLPNO-CCSD(T)/def2-TZVP(CPCM(C),THF)// M06-2X/cc-pVDZ(pcm,THF): -1646.507279

TS 3d -> 4d + PPh_3
M06-2X/cc-pVDZ(pcm,THF): -1648.797803
DLPNO-CCSD(T)/def2-TZVP(CPCM(C),THF)// M06-2X/cc-pVDZ(pcm,THF): -1646.461321

Cyclopropane 4d
M06-2X/cc-pVDZ(pcm,THF): -612.7770328
DLPNO-CCSD(T)/def2-TZVP(CPCM(C),THF)// M06-2X/cc-pVDZ(pcm,THF): -611.987898

TS 3d -> 5d + PPh_3
M06-2X/cc-pVDZ(pcm,THF): -1648.7761239
DLPNO-CCSD(T)/def2-TZVP(CPCM(C),THF)// M06-2X/cc-pVDZ(pcm,THF): -1646.444489

Dihydrofuran 5d
M06-2X/cc-pVDZ(pcm,THF): -612.7651978
DLPNO-CCSD(T)/def2-TZVP(CPCM(C),THF)// M06-2X/cc-pVDZ(pcm,THF): -611.9761730

TS 1c + 2B -> 10d (Z)
M06-2X/cc-pVDZ(pcm,THF): -1648.7687431
DLPNO-CCSD(T)/def2-TZVP(COSMO,THF)// M06-2X/cc-pVDZ(pcm,THF): -1646.443156

Oxaphosphetane 10d (Z)
M06-2X/cc-pVDZ(pcm,THF): -1648.7990663
DLPNO-CCSD(T)/def2-TZVP(CPCM(C),THF)// M06-2X/cc-pVDZ(pcm,THF): -1646.479439

TS 10d (cis) -> 11d (Z) + O=PPh_3
M06-2X/cc-pVDZ(pcm,THF): -1648.7393911
DLPNO-CCSD(T)/def2-TZVP(CPCM(C),THF)// M06-2X/cc-pVDZ(pcm,THF): -1646.425970

Diene 11d (Z)
M06-2X/cc-pVDZ(pcm,THF): -537.512280
DLPNO-CCSD(T)/def2-TZVP(CPCM(C),THF)// M06-2X/cc-pVDZ(pcm,THF): -536.804911

TS 1c + 2B -> 10d (E)
M06-2X/cc-pVDZ(pcm,THF): -1648.7660571
DLPNO-CCSD(T)/def2-TZVP(CPCM(C),THF)// M06-2X/cc-pVDZ(pcm,THF): -1646.440923

Oxaphosphetane 10d (E)
M06-2X/cc-pVDZ(pcm,THF): -1648.801236
DLPNO-CCSD(T)/def2-TZVP(CPCM(C),THF)// M06-2X/cc-pVDZ(pcm,THF): -1646.482627

TS 10d (E) -> 11d + O=PPh_3
M06-2X/cc-pVDZ(pcm,THF): -1648.7467991
DLPNO-CCSD(T)/def2-TZVP(CPCM(C),THF)// M06-2X/cc-pVDZ(pcm,THF): -1646.435456

Diene 11d (E)
M06-2X/cc-pVDZ(pcm,THF): -537.513779
DLPNO-CCSD(T)/def2-TZVP(CPCM(C),THF)// M06-2X/cc-pVDZ(pcm,THF): -536.805643

TS 1c + 2C -> 3e
M06-2X/cc-pVDZ(pcm,THF): -1687.636017
DLPNO-CCSD(T)/def2-TZVP(CPCM(C),THF)// M06-2X/cc-pVDZ(pcm,THF): -1685.686774

Zwitterion 3e
M06-2X/cc-pVDZ(pcm,THF): -1688.1265884
DLPNO-CCSD(T)/def2-TZVP(CPCM(C),THF)// M06-2X/cc-pVDZ(pcm,THF): -1685.738641

TS 3e -> 4e + PPh_3
M06-2X/cc-pVDZ(pcm,THF): -1688.0894943
DLPNO-CCSD(T)/def2-TZVP(CPCM(C),THF)// M06-2X/cc-pVDZ(pcm,THF): -1685.693822

Cyclopropane 4e
M06-2X/cc-pVDZ(pcm,THF): -652.0729026
DLPNO-CCSD(T)/def2-TZVP(CPCM(C),THF)// M06-2X/cc-pVDZ(pcm,THF): -651.223550

TS 3e -> 5e + PPh_3
M06-2X/cc-pVDZ(pcm,THF): -1688.0776275
DLPNO-CCSD(T)/def2-TZVP(CPCM(C),THF)// M06-2X/cc-pVDZ(pcm,THF): -1685.678302

Dihydrofuran 5e
M06-2X/cc-pVDZ(pcm,THF): -652.0672093
DLPNO-CCSD(T)/def2-TZVP(CPCM(C),THF)// M06-2X/cc-pVDZ(pcm,THF): -651.219132

TS 1c + 2C -> 10e
M06-2X/cc-pVDZ(pcm,THF): -1688.056118
DLPNO-CCSD(T)/def2-TZVP(CPCM(C),THF)// M06-2X/cc-pVDZ(pcm,THF): -1685.669802

Oxaphosphetane 10e
M06-2X/cc-pVDZ(pcm,THF): -1688.0978791
DLPNO-CCSD(T)/def2-TZVP(CPCM(C),THF)// M06-2X/cc-pVDZ(pcm,THF): -1685.715551

TS 10e -> 11e + O=PPh_3
M06-2X/cc-pVDZ(pcm,THF): -1688.0430398
DLPNO-CCSD(T)/def2-TZVP(CPCM(C),THF)// M06-2X/cc-pVDZ(pcm,THF): -1685.670196

Diene 11e
M06-2X/cc-pVDZ(pcm,THF): -576.8099202
DLPNO-CCSD(T)/def2-TZVP(CPCM(C),THF)// M06-2X/cc-pVDZ(pcm,THF): -576.039969

Methyl acrylate 1f
M06-2X/cc-pVDZ(pcm,THF): -306.3665028
DLPNO-CCSD(T)/def2-TZVP(CPCM(C),THF)// M06-2X/cc-pVDZ(pcm,THF): -305.978450

TS 1f + 2A -> 3f
M06-2X/cc-pVDZ(pcm,THF): -1381.6687489
DLPNO-CCSD(T)/def2-TZVP(CPCM(C),THF)// M06-2X/cc-pVDZ(pcm,THF): -1379.667766

Zwitterion 3f
M06-2X/cc-pVDZ(pcm,THF): -1381.6926098
DLPNO-CCSD(T)/def2-TZVP(CPCM(C),THF)// M06-2X/cc-pVDZ(pcm,THF): -1379.701673

Phosphacycle 3f’
M06-2X/cc-pVDZ(pcm,THF): -1381.6938798
DLPNO-CCSD(T)/def2-TZVP(CPCM(C),THF)// M06-2X/cc-pVDZ(pcm,THF): -1379.703439

TS 3f -> 4f + PPh_3
M06-2X/cc-pVDZ(pcm,THF): -1381.6642281
DLPNO-CCSD(T)/def2-TZVP(CPCM(C),THF)// M06-2X/cc-pVDZ(pcm,THF): -1379.663939

Methyl cyclopropanecarboxylate 4f
M06-2X/cc-pVDZ(pcm,THF): -345.6622788
DLPNO-CCSD(T)/def2-TZVP(CPCM(C),THF)// M06-2X/cc-pVDZ(pcm,THF): -345.206319

TS 3f -> 5f + PPh_3
M06-2X/cc-pVDZ(pcm,THF): -1381.6380707
DLPNO-CCSD(T)/def2-TZVP(CPCM(C),THF)// M06-2X/cc-pVDZ(pcm,THF): -1379.642213

4*H*,5*H*-2-Methoxyfuran 5f
M06-2X/cc-pVDZ(pcm,THF): -345.6325229
DLPNO-CCSD(T)/def2-TZVP(CPCM(C),THF)// M06-2X/cc-pVDZ(pcm,THF): -345.177901

TS 1f + 2A -> 10f
M06-2X/cc-pVDZ(pcm,THF): -1381.657953
DLPNO-CCSD(T)/def2-TZVP(CPCM(C),THF)// M06-2X/cc-pVDZ(pcm,THF): -1379.663285

Oxaphosphetane 10f
M06-2X/cc-pVDZ(pcm,THF): -1381.6819112
DLPNO-CCSD(T)/def2-TZVP(CPCM(C),THF)// M06-2X/cc-pVDZ(pcm,THF): -1379.691994

TS 10f -> 11f + $O=PPh_3$
M06-2X/cc-pVDZ(pcm,THF): -1381.6380894
DLPNO-CCSD(T)/def2-TZVP(CPCM(C),THF)// M06-2X/cc-pVDZ(pcm,THF): -1379.655004

2-Methoxy-1,3-butadiene 11f
M06-2X/cc-pVDZ(pcm,THF): -270.4102807

DLPNO-CCSD(T)/def2-TZVP(CPCM(C),THF)// M06-2X/cc-pVDZ(pcm,THF): -270.034810
Dimethylbenzylidenemalonate 1g
M06-2X/cc-pVDZ(pcm,THF): -765.1697012
DLPNO-CCSD(T)/def2-TZVP(CPCM(C),THF)// M06-2X/cc-pVDZ(pcm,THF): -764.131118

TS 1g + 2A -> 3g
M06-2X/cc-pVDZ(pcm,THF): -1840.4704715
DLPNO-CCSD(T)/def2-TZVP(CPCM(C),THF)// M06-2X/cc-pVDZ(pcm,THF): -1837.829879

Zwitterion 3g
M06-2X/cc-pVDZ(pcm,THF): -1840.5225128
DLPNO-CCSD(T)/def2-TZVP(CPCM(C),THF)// M06-2X/cc-pVDZ(pcm,THF): -1837.882485

TS 3g -> 4g + PPh_3
M06-2X/cc-pVDZ(pcm,THF): -1840.4838853
DLPNO-CCSD(T)/def2-TZVP(CPCM(C),THF)// M06-2X/cc-pVDZ(pcm,THF): -1837.833639

Cyclopropane 4g
M06-2X/cc-pVDZ(pcm,THF): -804.466289
DLPNO-CCSD(T)/def2-TZVP(CPCM(C),THF)// M06-2X/cc-pVDZ(pcm,THF): -803.361537

TS 3g -> 5g + PPh_3
M06-2X/cc-pVDZ(pcm,THF): -1840.4708242
DLPNO-CCSD(T)/def2-TZVP(CPCM(C),THF)// M06-2X/cc-pVDZ(pcm,THF): -1837.825541

Dihydrofuran 5g
M06-2X/cc-pVDZ(pcm,THF): -804.4502809
DLPNO-CCSD(T)/def2-TZVP(CPCM(C),THF)// M06-2X/cc-pVDZ(pcm,THF): -803.345496

TS 1g + 2A -> 10g
M06-2X/cc-pVDZ(pcm,THF): -1840.4697971
DLPNO-CCSD(T)/def2-TZVP(CPCM(C),THF)// M06-2X/cc-pVDZ(pcm,THF): -1837.832262

Oxaphosphetane 10g
M06-2X/cc-pVDZ(pcm,THF): -1840.4880433
DLPNO-CCSD(T)/def2-TZVP(CPCM(C),THF)// M06-2X/cc-pVDZ(pcm,THF): -1837.851788

TS 10g -> 11g + $O{=}PPh_3$
M06-2X/cc-pVDZ(pcm,THF): -1840.4379081
DLPNO-CCSD(T)/def2-TZVP(CPCM(C),THF)// M06-2X/cc-pVDZ(pcm,THF): -1837.812212

E-1-Phenyl-3-methoxy-1,3-butadiene-2-carboxylic acid methyl ester 11g
M06-2X/cc-pVDZ(pcm,THF): -729.2067572
DLPNO-CCSD(T)/def2-TZVP(CPCM(C),THF)// M06-2X/cc-pVDZ(pcm,THF): -728.1820156

TS 1g + 2C -> 3h
M06-2X/cc-pVDZ(pcm,THF): -1919.0589706
DLPNO-CCSD(T)/def2-TZVP(CPCM(C),THF)// M06-2X/cc-pVDZ(pcm,THF): -1916.296899

Zwitterion 3h
M06-2X/cc-pVDZ(pcm,THF): -1919.1020643

DLPNO-CCSD(T)/def2-TZVP(CPCM(C),THF)// M06-2X/cc-pVDZ(pcm,THF): -1916.339163

TS 3h -> 4h + PPh_3
M06-2X/cc-pVDZ(pcm,THF): -1919.0724916
DLPNO-CCSD(T)/def2-TZVP(CPCM(C),THF)// M06-2X/cc-pVDZ(pcm,THF): -1916.298246

Cyclopropane 4h
M06-2X/cc-pVDZ(pcm,THF): -883.0571449
DLPNO-CCSD(T)/def2-TZVP(CPCM(C),THF)// M06-2X/cc-pVDZ(pcm,THF): -881.831472

TS 3h -> 5h + PPh_3
M06-2X/cc-pVDZ(pcm,THF): -1919.0643917
DLPNO-CCSD(T)/def2-TZVP(CPCM(C),THF)// M06-2X/cc-pVDZ(pcm,THF): -1916.290922

Dihydrofuran 5h
M06-2X/cc-pVDZ(pcm,THF): -883.0531032
DLPNO-CCSD(T)/def2-TZVP(CPCM(C),THF)// M06-2X/cc-pVDZ(pcm,THF): -881.830692

TS 1g + 2C -> 10h
M06-2X/cc-pVDZ(pcm,THF): -1919.0475293
DLPNO-CCSD(T)/def2-TZVP(CPCM(C),THF)// M06-2X/cc-pVDZ(pcm,THF): -1916.287702

Oxaphosphetane 10h
M06- 2X/cc-pVDZ(pcm,THF): -1919.0825152
DLPNO-CCSD(T)/def2-TZVP(CPCM(C),THF)// M06-2X/cc-pVDZ(pcm,THF): -1916.326098

TS 10h -> 11h + O=PPh_3
M06- 2X/cc-pVDZ(pcm,THF): -1919.0159053
DLPNO-CCSD(T)/def2-TZVP(CPCM(C),THF)// M06-2X/cc-pVDZ(pcm,THF): -1916.267143

Diene 11h
M06- 2X/cc-pVDZ(pcm,THF): -807.7959612
DLPNO-CCSD(T)/def2-TZVP(CPCM(C),THF)// M06-2X/cc-pVDZ(pcm,THF): -806.649180

Dimethyl-4-methoxybenzylidenemalonate 1i
M06-2X/cc-pVDZ(pcm,THF): -879.6608907
DLPNO-CCSD(T)/def2-TZVP(CPCM(C),THF)// M06-2X/cc-pVDZ(pcm,THF): -878.484189

TS 1i + 2A -> 3i
M06-2X/cc-pVDZ(pcm,THF): -1954.9607274
DLPNO-CCSD(T)/def2-TZVP(CPCM(C),THF)// M06-2X/cc-pVDZ(pcm,THF): -1952.183048

Zwitterion 3i
M06-2X/cc-pVDZ(pcm,THF): -1955.0119342
DLPNO-CCSD(T)/def2-TZVP(CPCM(C),THF)// M06-2X/cc-pVDZ(pcm,THF): -1952.234344

TS 3i -> 4i + PPh_3
M06-2X/cc-pVDZ(pcm,THF): -1954.9738758
DLPNO-CCSD(T)/def2-TZVP(CPCM(C),THF)// M06-2X/cc-pVDZ(pcm,THF): -1952.187543

Cyclopropane 4i

M06-2X/cc-pVDZ(pcm,THF): -918.9559025
DLPNO-CCSD(T)/def2-TZVP(CPCM(C),THF)// M06-2X/cc-pVDZ(pcm,THF): -917.713649

TS 3i -> 5i + PPh_3
M06-2X/cc-pVDZ(pcm,THF): -1954.9603774
DLPNO-CCSD(T)/def2-TZVP(CPCM(C),THF)// M06-2X/cc-pVDZ(pcm,THF): -1952.178407

Dihydrofuran 5i
M06-2X/cc-pVDZ(pcm,THF): -918.9394166
DLPNO-CCSD(T)/def2-TZVP(CPCM(C),THF)// M06-2X/cc-pVDZ(pcm,THF): -917.697179

TS 1i + 2A -> 10i
M06-2X/cc-pVDZ(pcm,THF): -1954.9600168
DLPNO-CCSD(T)/def2-TZVP(CPCM(C),THF)// M06-2X/cc-pVDZ(pcm,THF): -1952.184717

Oxaphosphetane 10i
M06-2X/cc-pVDZ(pcm,THF): -1954.978136
DLPNO-CCSD(T)/def2-TZVP(CPCM(C),THF)// M06-2X/cc-pVDZ(pcm,THF): -1952.204040
TS 10i -> 11i + O=PPh_3
M06-2X/cc-pVDZ(pcm,THF): -1954.9282668
DLPNO-CCSD(T)/def2-TZVP(CPCM(C),THF)// M06-2X/cc-pVDZ(pcm,THF): -1952.164688

1-(4-Methoxyphenyl)-3-methoxy-1,3-butadiene-2-carboxylic acid methyl ester 11i
M06-2X/cc-pVDZ(pcm,THF): -843.6977491
DLPNO-CCSD(T)/def2-TZVP(CPCM(C),THF)// M06-2X/cc-pVDZ(pcm,THF): -842.534809

TS 1i + 2C -> 3j
M06-2X/cc-pVDZ(pcm,THF): -2033.5494472
DLPNO-CCSD(T)/def2-TZVP(CPCM(C),THF)// M06-2X/cc-pVDZ(pcm,THF): -2030.650210

Zwitterion 3j
M06-2X/cc-pVDZ(pcm,THF): -2033.5919202
DLPNO-CCSD(T)/def2-TZVP(CPCM(C),THF)// M06-2X/cc-pVDZ(pcm,THF): -2030.689915

TS 3j -> 4j + PPh_3
M06-2X/cc-pVDZ(pcm,THF): -2033.5628701
DLPNO-CCSD(T)/def2-TZVP(CPCM(C),THF)// M06-2X/cc-pVDZ(pcm,THF): -2030.652040

Cyclopropane 4j
M06-2X/cc-pVDZ(pcm,THF): -997.5465248
DLPNO-CCSD(T)/def2-TZVP(CPCM(C),THF)// M06-2X/cc-pVDZ(pcm,THF): -996.183290

TS 3j -> 5j + PPh_3
M06-2X/cc-pVDZ(pcm,THF): -2033.5536598
DLPNO-CCSD(T)/def2-TZVP(CPCM(C),THF)// M06-2X/cc-pVDZ(pcm,THF): -2030.643031

Dihydrofuran 5j
M06-2X/cc-pVDZ(pcm,THF): -997.5422549
DLPNO-CCSD(T)/def2-TZVP(CPCM(C),THF)// M06-2X/cc-pVDZ(pcm,THF): -996.182209

TS 1i + 2C -> 10j

M06-2X/cc-pVDZ(pcm,THF): -2033.5376384
DLPNO-CCSD(T)/def2-TZVP(CPCM(C),THF)// M06-2X/cc-pVDZ(pcm,THF): -2030.640509
Oxaphosphetane 10j
M06-2X/cc-pVDZ(pcm,THF): -2033.5722899
DLPNO-CCSD(T)/def2-TZVP(CPCM(C),THF)// M06-2X/cc-pVDZ(pcm,THF): -2030.678346

TS 10j -> 11j + O=PPh_3
M06-2X/cc-pVDZ(pcm,THF): -2033.5057394
DLPNO-CCSD(T)/def2-TZVP(CPCM(C),THF)// M06-2X/cc-pVDZ(pcm,THF): -2030.619300

Diene 11j
M06-2X/cc-pVDZ(pcm,THF): -922.2865622
DLPNO-CCSD(T)/def2-TZVP(CPCM(C),THF)// M06-2X/cc-pVDZ(pcm,THF): -921.002029

Dimethyl-(4-nitrobenzylidene)malonate 1k
M06-2X/cc-pVDZ(pcm,THF): -969.6249443
DLPNO-CCSD(T)/def2-TZVP(CPCM(C),THF)// M06-2X/cc-pVDZ(pcm,THF): -968.370182

TS 1k + 2A -> 3k
M06-2X/cc-pVDZ(pcm,THF): -2044.9313397
DLPNO-CCSD(T)/def2-TZVP(CPCM(C),THF)// M06-2X/cc-pVDZ(pcm,THF): -2042.076849

Zwitterion 3k
M06-2X/cc-pVDZ(pcm,THF): -2044.9820874
DLPNO-CCSD(T)/def2-TZVP(CPCM(C),THF)// M06-2X/cc-pVDZ(pcm,THF): -2042.124155

TS 3k -> 4k + PPh_3
M06-2X/ cc-pVDZ(pcm,THF): -2044.94445
DLPNO-CCSD(T)/def2-TZVP(CPCM(C),THF)// M06-2X/cc-pVDZ(pcm,THF): -2042.074978

Cyclopropane 4k
M06-2X/cc-pVDZ(pcm,THF): -1008.9234723
DLPNO-CCSD(T)/def2-TZVP(CPCM(C),THF)// M06-2X/cc-pVDZ(pcm,THF): -1007.601722

TS 3k -> 5k + PPh_3
M06-2X/cc-pVDZ(pcm,THF): -2044.9322192
DLPNO-CCSD(T)/def2-TZVP(CPCM(C),THF)// M06-2X/cc-pVDZ(pcm,THF): -2042.070209

Dihydrofuran 5k
M06-2X/cc-pVDZ(pcm,THF): -1008.9089308
DLPNO-CCSD(T)/def2-TZVP(CPCM(C),THF)// M06-2X/cc-pVDZ(pcm,THF): -1007.586618

TS 1k + 2A -> 10k
M06-2X/cc-pVDZ(pcm,THF): -2044.9281228
DLPNO-CCSD(T)/def2-TZVP(CPCM(C),THF)// M06-2X/cc-pVDZ(pcm,THF): -2042.073683

Oxaphosphetane 10k
M06-2X/cc-pVDZ(pcm,THF): -2044.9458246
DLPNO-CCSD(T)/def2-TZVP(CPCM(C),THF)// M06-2X/cc-pVDZ(pcm,THF): -2042.092573

TS 10 k -> 11k + O=PPh_3

M06-2X/cc-pVDZ(pcm,THF): -2044.8945458
DLPNO-CCSD(T)/def2-TZVP(CPCM(C),THF)// M06-2X/cc-pVDZ(pcm,THF): -2042.0520778

1-(4-Nitrophenyl)-3-methoxy-1,3-butadiene-2-carboxylic acid methyl ester 11k
M06-2X/cc-pVDZ(pcm,THF): -933.6631515
DLPNO-CCSD(T)/def2-TZVP(CPCM(C),THF)// M06-2X/cc-pVDZ(pcm,THF): -932.422325

TS 1k + 2C -> 3l
M06-2X/cc-pVDZ(pcm,THF): -2123.5182868
DLPNO-CCSD(T)/def2-TZVP(CPCM(C),THF)// M06-2X/cc-pVDZ(pcm,THF): -2120.539756

Zwitterion 3l
M06-2X/cc-pVDZ(pcm,THF): -2123.5614381
DLPNO-CCSD(T)/def2-TZVP(CPCM(C),THF)// M06-2X/cc-pVDZ(pcm,THF): -2120.581245

TS 3l -> 4l + PPh_3
M06-2X/cc-pVDZ(pcm,THF): -2123.5312573
DLPNO-CCSD(T)/def2-TZVP(CPCM(C),THF)// M06-2X/cc-pVDZ(pcm,THF): -2120.540867

Cyclopropane 4l
M06-2X/cc-pVDZ(pcm,THF): -1087.5146097
DLPNO-CCSD(T)/def2-TZVP(CPCM(C),THF)// M06-2X/cc-pVDZ(pcm,THF): -1086.072097

TS 3l -> 5l + PPh_3
M06-2X/cc-pVDZ(pcm,THF): -2123.5247924
DLPNO-CCSD(T)/def2-TZVP(CPCM(C),THF)// M06-2X/cc-pVDZ(pcm,THF): -2120.534194

Dihydrofuran 5l
M06-2X/cc-pVDZ(pcm,THF): -1087.5115505
DLPNO-CCSD(T)/def2-TZVP(CPCM(C),THF)// M06-2X/cc-pVDZ(pcm,THF): -1086.071820

TS 1k + 2C -> 10l
M06-2X/cc-pVDZ(pcm,THF): -2123.5059371
DLPNO-CCSD(T)/def2-TZVP(CPCM(C),THF)// M06-2X/cc-pVDZ(pcm,THF): -2120.529682

Oxaphosphetane 10l
M06-2X/cc-pVDZ(pcm,THF): -2123.540442
DLPNO-CCSD(T)/def2-TZVP(CPCM(C),THF)// M06-2X/cc-pVDZ(pcm,THF): -2120.566454

TS 10l -> 11l + O=PPh_3
M06-2X/cc-pVDZ(pcm,THF): -2123.4734319
DLPNO-CCSD(T)/def2-TZVP(CPCM(C),THF)// M06-2X/cc-pVDZ(pcm,THF): -2120.507556

Diene 11l
M06-2X/cc-pVDZ(pcm,THF): -1012.2525979
DLPNO-CCSD(T)/def2-TZVP(CPCM(C),THF)// M06-2X/cc-pVDZ(pcm,THF): -1010.889691

N,N,N′,N′-Tetramethylmethylenemalondiamide 1m
M06-2X/cc-pVDZ(pcm,THF): -573.0326791
DLPNO-CCSD(T)/def2-TZVP(CPCM(C),THF)// M06-2X/cc-pVDZ(pcm,THF): -572.259418

TS 1m + 2A -> 3m

M06-2X/cc-pVDZ(pcm,THF): -1648.3298644
DLPNO-CCSD(T)/def2-TZVP(CPCM(C),THF)// M06-2X/cc-pVDZ(pcm,THF): -1645.956068

Zwitterion 3m
M06-2X/cc-pVDZ(pcm,THF): -1648.3723184
DLPNO-CCSD(T)/def2-TZVP(CPCM(C),THF)// M06-2X/cc-pVDZ(pcm,THF): -1645.995536

TS 3m -> 4m + PPh_3
M06-2X/cc-pVDZ(pcm,THF): -1648.3394992
DLPNO-CCSD(T)/def2-TZVP(CPCM(C),THF)// M06-2X/cc-pVDZ(pcm,THF): -1645.954555

Cyclopropane 4m
M06-2X/cc-pVDZ(pcm,THF): -612.3324637
DLPNO-CCSD(T)/def2-TZVP(CPCM(C),THF)// M06-2X/cc-pVDZ(pcm,THF): -611.490782

TS 3m -> 5m + PPh_3
M06-2X/cc-pVDZ(pcm,THF): -1648.3203095
DLPNO-CCSD(T)/def2-TZVP(CPCM(C),THF)// M06-2X/cc-pVDZ(pcm,THF): -1645.938316

Dihydrofuran 5m
M06-2X/cc-pVDZ(pcm,THF): -612.3123407
DLPNO-CCSD(T)/def2-TZVP(CPCM(C),THF)// M06-2X/cc-pVDZ(pcm,THF): -611.473477

TS 1m + 2A -> 10m
M06-2X/cc-pVDZ(pcm,THF): -1648.3184331
DLPNO-CCSD(T)/def2-TZVP(CPCM(C),THF)// M06-2X/cc-pVDZ(pcm,THF): -1645.944190

Oxaphosphetane 10m
M06-2X/cc-pVDZ(pcm,THF): -1648.3453206
DLPNO-CCSD(T)/def2-TZVP(CPCM(C),THF)// M06-2X/cc-pVDZ(pcm,THF): -1645.978539

TS 10m -> 11m + $OPPh_3$
M06-2X/cc-pVDZ(pcm,THF): -1648.3068148
DLPNO-CCSD(T)/def2-TZVP(CPCM(C),THF)// M06-2X/cc-pVDZ(pcm,THF): -1645.949121

Diene 11m
M06-2X/cc-pVDZ(pcm,THF): -537.0718341
DLPNO-CCSD(T)/def2-TZVP(CPCM(C),THF)// M06-2X/cc-pVDZ(pcm,THF): -536. 313641

Methyl cyanoacrylate 1n
M06-2X/cc-pVDZ(pcm,THF): -398.5936256
DLPNO-CCSD(T)/def2-TZVP(CPCM(C),THF)// M06-2X/cc-pVDZ(pcm,THF): -398.076076

TS 1n + 2A -> 3n
M06-2X/cc-pVDZ(pcm,THF): -1473.8992314
DLPNO-CCSD(T)/def2-TZVP(CPCM(C),THF)// M06-2X/cc-pVDZ(pcm,THF): -1471.780224

Zwitterion 3n
M06-2X/cc-pVDZ(pcm,THF): -1473.9524278
DLPNO-CCSD(T)/def2-TZVP(CPCM(C),THF)// M06-2X/cc-pVDZ(pcm,THF): -1471.834553

TS 3n -> 4n + PPh_3
M06-2X/cc-pVDZ(pcm,THF): -1473.9148321
DLPNO-CCSD(T)/def2-TZVP(CPCM(C),THF)// M06-2X/cc-pVDZ(pcm,THF): -1471.784457

Cyclopropane 4n
M06-2X/cc-pVDZ(pcm,THF): -437.8908408
DLPNO-CCSD(T)/def2-TZVP(CPCM(C),THF)// M06-2X/cc-pVDZ(pcm,THF): -437.306875

TS 3n -> 5n + PPh_3
M06-2X/cc-pVDZ(pcm,THF): -1473.8943283
DLPNO-CCSD(T)/def2-TZVP(CPCM(C),THF)// M06-2X/cc-pVDZ(pcm,THF): -1471.767166

Dihydrofuran 5n
M06-2X/cc-pVDZ(pcm,THF): -437.8758767
DLPNO-CCSD(T)/def2-TZVP(CPCM(C),THF)// M06-2X/cc-pVDZ(pcm,THF): -437.291521
1,1-Dicyanoethene 1o
M06-2X/cc-pVDZ(pcm,THF): -262.9964145
DLPNO-CCSD(T)/def2-TZVP(CPCM(C),THF)// M06-2X/cc-pVDZ(pcm,THF): -262.624046

TS 1o + 2A -> 3o
M06-2X/cc-pVDZ(pcm,THF): -1338.3032947
DLPNO-CCSD(T)/def2-TZVP(CPCM(C),THF)// M06-2X/cc-pVDZ(pcm,THF): -1336.333113

Zwitterion 3o
M06-2X/cc-pVDZ(pcm,THF): -1338.3591996
DLPNO-CCSD(T)/def2-TZVP(CPCM(C),THF)// M06-2X/cc-pVDZ(pcm,THF): -1336.384824

TS 3o -> 4o + PPh_3
M06-2X/cc-pVDZ(pcm,THF): -1338.3168251
DLPNO-CCSD(T)/def2-TZVP(CPCM(C),THF)// M06-2X/cc-pVDZ(pcm,THF): -1336.327458

1,1-Dicyanocyclopropane 4o
M06-2X/cc-pVDZ(pcm,THF): -302.2919095
DLPNO-CCSD(T)/def2-TZVP(CPCM(C),THF)// M06-2X/cc-pVDZ(pcm,THF): -301.853365

Acrylonitrile 1p
M06-2X/cc-pVDZ(pcm,THF): -170.7778903
DLPNO-CCSD(T)/def2-TZVP(CPCM(C),THF)// M06-2X/cc-pVDZ(pcm,THF): -170.534202

TS 1p + 2A -> 3p
M06-2X/cc-pVDZ(pcm,THF): -1246.0802521
DLPNO-CCSD(T)/def2-TZVP(CPCM(C),THF)// M06-2X/cc-pVDZ(pcm,THF): -1244.238718

Zwitterion 3p
M06-2X/cc-pVDZ(pcm,THF): -1246.0955098
DLPNO-CCSD(T)/def2-TZVP(CPCM(C),THF)// M06-2X/cc-pVDZ(pcm,THF): -1244.251921

TS 3p -> 4p + PPh_3
M06-2X/cc-pVDZ(pcm,THF): -1246.0708118
DLPNO-CCSD(T)/def2-TZVP(CPCM(C),THF)// M06-2X/cc-pVDZ(pcm,THF): -1244.217570

Cyanocyclopropane 4p
M06-2X/cc-pVDZ(pcm,THF): -210.0728651
DLPNO-CCSD(T)/def2-TZVP(CPCM(C),THF)// M06-2X/cc-pVDZ(pcm,THF): -209.761866

1,1-Dinitroethene 1q
M06-2X/cc-pVDZ(pcm,THF): -487.4344492
DLPNO-CCSD(T)/def2-TZVP(CPCM(C),THF)// M06-2X/cc-pVDZ(pcm,THF): -486.888472

The reaction 1q + 2A -> 3q is barrierless, no transition state

Zwitterion 3q
M06-2X/cc-pVDZ(pcm,THF): -1562.8281114
DLPNO-CCSD(T)/def2-TZVP(CPCM(C),THF)// M06-2X/cc-pVDZ(pcm,THF): -1560.675408

TS 3q -> 4q + PPh_3
M06-2X/cc-pVDZ(pcm,THF): -1562.7695165
DLPNO-CCSD(T)/def2-TZVP(CPCM(C),THF)// M06-2X/cc-pVDZ(pcm,THF): -1560.605623

1,1-Dinitrocyclopropane 4q
M06-2X/cc-pVDZ(pcm,THF): -526.738580
DLPNO-CCSD(T)/def2-TZVP(CPCM(C),THF)// M06-2X/cc-pVDZ(pcm,THF): -526.124269

TS 3q -> 6q + PPh_3
M06-2X/cc-pVDZ(pcm,THF): -1562.7649637
DLPNO-CCSD(T)/def2-TZVP(CPCM(C),THF)// M06-2X/cc-pVDZ(pcm,THF): -1560.607125

Cyclic nitrone 6q
M06-2X/cc-pVDZ(pcm,THF): -526.7425476
DLPNO-CCSD(T)/def2-TZVP(CPCM(C),THF)// M06-2X/cc-pVDZ(pcm,THF): -526.129968

Nitroethene 1r
M06-2X/cc-pVDZ(pcm,THF): -283.0026354
DLPNO-CCSD(T)/def2-TZVP(CPCM(C),THF)// M06-2X/cc-pVDZ(pcm,THF): -282.671861

TS 1r + 2A -> 3r
M06-2X/cc-pVDZ(pcm,THF): -1358.3038828
DLPNO-CCSD(T)/def2-TZVP(CPCM(C),THF)// M06-2X/cc-pVDZ(pcm,THF): -1356.370358

Zwitterion 3r
M06-2X/cc-pVDZ(pcm,THF): -1358.3552484
DLPNO-CCSD(T)/def2-TZVP(CPCM(C),THF)// M06-2X/cc-pVDZ(pcm,THF): -1356.424938

TS 3r -> 4r + PPh_3
M06-2X/cc-pVDZ(pcm,THF): -1358.3146576
DLPNO-CCSD(T)/def2-TZVP(CPCM(C),THF)// M06-2X/cc-pVDZ(pcm,THF): -1356.370749

Nitrocyclopropane 4r
M06-2X/cc-pVDZ(pcm,THF): -322.3018377
DLPNO-CCSD(T)/def2-TZVP(CPCM(C),THF)// M06-2X/cc-pVDZ(pcm,THF): -321.902525

TS 3r -> 6r + PPh_3
M06-2X/cc-pVDZ(pcm,THF): -1358.3037531
DLPNO-CCSD(T)/def2-TZVP(CPCM(C),THF)// M06-2X/cc-pVDZ(pcm,THF): -1356.368290

Cyclic nitrone 6r
M06-2X/cc-pVDZ(pcm,THF): -322.298430
DLPNO-CCSD(T)/def2-TZVP(CPCM(C),THF)// M06-2X/cc-pVDZ(pcm,THF): -321.905064

Phosphonate 1s
M06-2X/cc-pVDZ(pcm,THF): -952.5491102
DLPNO-CCSD(T)/def2-TZVP(CPCM(C),THF)// M06-2X/cc-pVDZ(pcm,THF): -951.433578

TS 1s + 2A -> 3s
M06-2X/cc-pVDZ(pcm,THF): -2027.8535087
DLPNO-CCSD(T)/def2-TZVP(CPCM(C),THF)// M06-2X/cc-pVDZ(pcm,THF): -2025.138393
Zwitterion 3s
M06-2X/cc-pVDZ(pcm,THF): -2027.907559
DLPNO-CCSD(T)/def2-TZVP(CPCM(C),THF)// M06-2X/cc-pVDZ(pcm,THF): -2025.187735

TS 3s -> 4s + PPh_3
M06-2X/cc-pVDZ(pcm,THF): -2027.8679404
DLPNO-CCSD(T)/def2-TZVP(CPCM(C),THF)// M06-2X/cc-pVDZ(pcm,THF): -2025.141103

Cyclopropane 4s
M06-2X/cc-pVDZ(pcm,THF): -991.847825
DLPNO-CCSD(T)/def2-TZVP(CPCM(C),THF)// M06-2X/cc-pVDZ(pcm,THF): -990.666940

TS 3s -> 5s + PPh_3
M06-2X/cc-pVDZ(pcm,THF): -2027.8472036
DLPNO-CCSD(T)/def2-TZVP(CPCM(C),THF)// M06-2X/cc-pVDZ(pcm,THF): -2025.121740

Dihydrofuran 5s
M06-2X/cc-pVDZ(pcm,THF): -991.8286907
DLPNO-CCSD(T)/def2-TZVP(CPCM(C),THF)// M06-2X/cc-pVDZ(pcm,THF): -990.649412

TS 3s -> 7s + PPh_3
M06-2X/cc-pVDZ(pcm,THF): -2027.8406428
DLPNO-CCSD(T)/def2-TZVP(CPCM(C),THF)// M06-2X/cc-pVDZ(pcm,THF): -2025.112083

Cyclic P-ylide 7s
M06-2X/cc-pVDZ(pcm,THF): -991.8169408
DLPNO-CCSD(T)/def2-TZVP(CPCM(C),THF)// M06-2X/cc-pVDZ(pcm,THF): -990.629737

1-Cyanoacrolein-*E*-*N*-phenylimine 1t
M06-2X/cc-pVDZ(pcm,THF): -495.1832896
DLPNO-CCSD(T)/def2-TZVP(CPCM(C),THF)// M06-2X/cc-pVDZ(pcm,THF): -494.431145

TS 1t + 2A -> 3t
M06-2X/cc-pVDZ(pcm,THF): -1570.484823
DLPNO-CCSD(T)/def2-TZVP(CPCM(C),THF)// M06-2X/cc-pVDZ(pcm,THF): -1568.133488

Zwitterion 3t
M06-2X/cc-pVDZ(pcm,THF): -1570.5494947
DLPNO-CCSD(T)/def2-TZVP(CPCM(C),THF)// M06-2X/cc-pVDZ(pcm,THF): -1568.189753

TS 3t -> 4t + PPh_3
M06-2X/cc-pVDZ(pcm,THF): -1570.5039171
DLPNO-CCSD(T)/def2-TZVP(CPCM(C),THF)// M06-2X/cc-pVDZ(pcm,THF): -1568.136920

Cyclopropane 4t
M06-2X/cc-pVDZ(pcm,THF): -534.4752096
DLPNO-CCSD(T)/def2-TZVP(CPCM(C),THF)// M06-2X/cc-pVDZ(pcm,THF): -533.657082

TS 3t -> 8t + PPh_3
M06-2X/cc-pVDZ(pcm,THF): -1570.4949329
DLPNO-CCSD(T)/def2-TZVP(CPCM(C),THF)// M06-2X/cc-pVDZ(pcm,THF): -1568.131727
4H,5H-3-Cyano-*N*-phenylpyrrole 8t
M06-2X/cc-pVDZ(pcm,THF): -534.5050655
DLPNO-CCSD(T)/def2-TZVP(CPCM(C),THF)// M06-2X/cc-pVDZ(pcm,THF): -533.684913

TS 1t + 2A -> 10t
M06-2X/cc-pVDZ(pcm,THF): -1570.4857944
DLPNO-CCSD(T)/def2-TZVP(CPCM(C),THF)// M06-2X/cc-pVDZ(pcm,THF): -1568.133403

Azaphosphetane 10t
M06-2X/cc-pVDZ(pcm,THF): -1570.5225185
DLPNO-CCSD(T)/def2-TZVP(CPCM(C),THF)// M06-2X/cc-pVDZ(pcm,THF): -1568.1757087

TS 10t -> 11t + PhN=PPh_3
M06-2X/cc-pVDZ(pcm,THF): -1570.4592264
DLPNO-CCSD(T)/def2-TZVP(CPCM(C),THF)// M06-2X/cc-pVDZ(pcm,THF): -1568.117154

2-Cyanobutadiene 11t
M06-2X/cc-pVDZ(pcm,THF): -248.151886
DLPNO-CCSD(T)/def2-TZVP(CPCM(C),THF)// M06-2X/cc-pVDZ(pcm,THF): -247.7855598

Methyldithiacyanoacrylate 1u
M06-2X/cc-pVDZ(pcm,THF): -1044.5135859
DLPNO-CCSD(T)/def2-TZVP(CPCM(C),THF)// M06-2X/cc-pVDZ(pcm,THF): -1043.256435

TS 1u + 2A -> 3u
M06-2X/cc-pVDZ(pcm,THF): -2119.8130971
DLPNO-CCSD(T)/def2-TZVP(CPCM(C),THF)// M06-2X/cc-pVDZ(pcm,THF): -2116.950316

Zwitterion 3u
M06-2X/cc-pVDZ(pcm,THF): -2119.8960874
DLPNO-CCSD(T)/def2-TZVP(CPCM(C),THF)// M06-2X/cc-pVDZ(pcm,THF): -2117.029674

TS 3u -> 4u + PPh_3
M06-2X/cc-pVDZ(pcm,THF): -2119.8449832
DLPNO-CCSD(T)/def2-TZVP(CPCM(C),THF)// M06-2X/cc-pVDZ(pcm,THF): -2116.968523

Cyclopropane 4u
M06-2X/cc-pVDZ(pcm,THF): -1083.8105069
DLPNO-CCSD(T)/def2-TZVP(CPCM(C),THF)// M06-2X/cc-pVDZ(pcm,THF): -1082.485164

TS 3u -> 9u + PPh_3
M06-2X/cc-pVDZ(pcm,THF): -2119.8462262
DLPNO-CCSD(T)/def2-TZVP(CPCM(C),THF)// M06-2X/cc-pVDZ(pcm,THF): -2116.972696

Dihydrothiophene 9u
M06-2X/cc-pVDZ(pcm,THF): -1083.8308474
DLPNO-CCSD(T)/def2-TZVP(CPCM(C),THF)// M06-2X/cc-pVDZ(pcm,THF): -1082.512653

TS 1u + 2A -> 11u + S=PPh_3
M06-2X/cc-pVDZ(pcm,THF): -2119.8136647
DLPNO-CCSD(T)/def2-TZVP(CPCM(C),THF)// M06-2X/cc-pVDZ(pcm,THF): -2116.9603566

TS 1u + 2A -> 10u'
M06-2X/cc-pVDZ(pcm,THF): -2119.8664589
DLPNO-CCSD(T)/def2-TZVP(CPCM(C),THF)// M06-2X/cc-pVDZ(pcm,THF): -2116.947798

Betain 10u'
M06-2X/cc-pVDZ(pcm,THF): -2119.881000
DLPNO-CCSD(T)/def2-TZVP(CPCM(C),THF)// M06-2X/cc-pVDZ(pcm,THF): -2117.015464

Diene 11u
M06-2X/cc-pVDZ(pcm,THF): -685.6172953
DLPNO-CCSD(T)/def2-TZVP(CPCM(C),THF)// M06-2X/cc-pVDZ(pcm,THF): -684.747623
